# EGR2 is an epigenomic regulator of phagocytosis and antifungal immunity in alveolar macrophages

**DOI:** 10.1172/jci.insight.164009

**Published:** 2024-09-10

**Authors:** Zsuzsanna Kolostyak, Dora Bojcsuk, Viktoria Baksa, Zsuzsa Mathene Szigeti, Krisztian Bene, Zsolt Czimmerer, Pal Boto, Lina Fadel, Szilard Poliska, Laszlo Halasz, Petros Tzerpos, Wilhelm K. Berger, Andres Villabona-Rueda, Zsofia Varga, Tunde Kovacs, Andreas Patsalos, Attila Pap, Gyorgy Vamosi, Peter Bai, Balazs Dezso, Matthew Spite, Franco R. D’Alessio, Istvan Szatmari, Laszlo Nagy

**Affiliations:** 1 Department of Biochemistry and Molecular Biology, Faculty of Medicine;; 2 Doctoral School of Molecular Cell and Immune Biology; and; 3 Department of Molecular Biotechnology and Microbiology, Faculty of Science, University of Debrecen, Debrecen, Hungary.; 4 Institute of Genetics, HUN-REN Biological Research Centre, Szeged, Szeged, Hungary.; 5 Department of Biophysics and Cell Biology, and; 6 Genomic Medicine and Bioinformatic Core Facility, Department of Biochemistry and Molecular Biology, Faculty of Medicine, University of Debrecen, Debrecen, Hungary.; 7 Departments of Medicine and Biological Chemistry, Johns Hopkins University School of Medicine, Institute for Fundamental Biomedical Research, Johns Hopkins All Children’s Hospital, St. Petersburg, Florida, USA.; 8 Division of Pulmonary Critical Care Medicine, Johns Hopkins University School of Medicine, Baltimore, Maryland, USA.; 9 Department of Medical Chemistry and; 10 Research Center for Molecular Medicine, Faculty of Medicine, University of Debrecen, Debrecen, Hungary.; 11 MTA-DE Cell Biology and Signaling Research Group ELKH, Debrecen, Hungary.; 12 Department of Pathology, Faculty of Medicine, and; 13 Department of Oral Pathology and Microbiology, Faculty of Dentistry, University of Debrecen, Debrecen, Hungary.; 14 Center for Experimental Therapeutics and Reperfusion Injury, Department of Anesthesiology, Perioperative and Pain Medicine, Brigham and Women’s Hospital, Harvard Medical School, Boston, Massachusetts, USA.

**Keywords:** Infectious disease, Inflammation, Fungal infections, Innate immunity, Transcription

## Abstract

Alveolar macrophages (AMs) act as gatekeepers of the lung’s immune responses, serving essential roles in recognizing and eliminating pathogens. The transcription factor (TF) early growth response 2 (EGR2) has been recently described as required for mature AMs in mice; however, its mechanisms of action have not been explored. Here, we identified EGR2 as an epigenomic regulator and likely direct proximal transcriptional activator in AMs using epigenomic approaches (RNA sequencing, ATAC sequencing, and CUT&RUN). The predicted direct proximal targets of EGR2 included a subset of AM identity genes and ones related to pathogen recognition, phagosome maturation, and adhesion, such as *Clec7a*, *Atp6v0d2*, *Itgb2*, *Rhoc*, and *Tmsb10*. We provided evidence that EGR2 deficiency led to impaired zymosan internalization and reduced the capacity to respond to *Aspergillus fumigatus*. Mechanistically, the lack of EGR2 altered the transcriptional response, secreted cytokines (i.e., CXCL11), and inflammation-resolving lipid mediators (i.e., RvE1) of AMs during in vivo zymosan-induced inflammation, which manifested in impaired resolution. Our findings demonstrated that EGR2 is a key proximal transcriptional activator and epigenomic bookmark in AMs responsible for select, distinct components of cell identity and a protective transcriptional and epigenomic program against fungi.

## Abstract 2

EGR2, a transcriptional and epigenomic regulator of alveolar macrophages controls late-stage markers and Dectin-1-mediated phagocytosis, modulates zymosan-induced inflammation and its absence results in prolonged inflammation.

## Graphical abstract

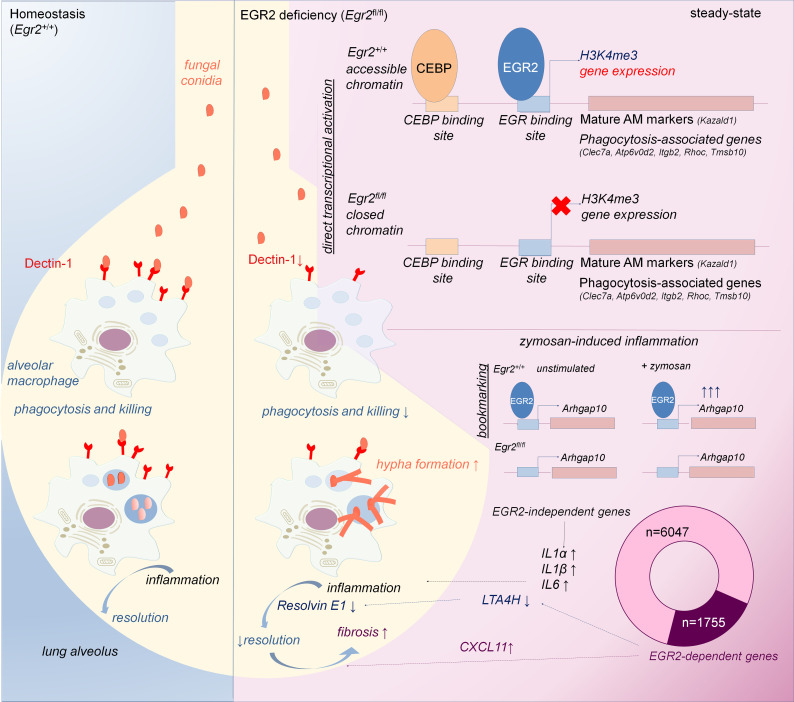

## Introduction

Macrophages are a heterogeneous population of cells, and their functions are regulated by microenvironment-dependent specific transcriptional regulators. The identity and molecular function of epigenomic regulators in alveolar macrophages (AMs) and in inflammatory lung pathologies are not well understood.

The resident AMs are localized in association with the bronchoalveolar epithelial lining, recognize and eliminate harmful agents, and initiate inflammatory responses. AMs identify pathogen-associated molecular patterns by pathogen recognition receptors (PRRs). The 2 main groups of PRRs are the Toll-like receptors (TLRs), which bind surface molecules specific for bacteria (1), and the C-type lectin receptors, such as Dectin-1, which recognize fungal cell wall components (2). After recognition, AMs phagocytose bound particles and inactivate them in phagolysosomes. The elimination processes are based on acidification, formation of reactive oxygen species (ROS) (3), and enzymatic lysis. Finally, AMs switch to a resolving phenotype and regenerate the damaged lung (4). Malfunctions of these processes lead to human diseases such as acute lung injury and pulmonary fibrosis.

AMs originate from the fetal liver. The fetal monocytes migrate to the fetal lung around embryonic day 13.5–14.5 (5, 6). In the fetal lung microenvironment, the presence of GM-CSF (7) and TGF-β (8) cytokines lead the differentiation to the pre-AM stage through the activation of purine rich box-1 (PU.1) (7), PPARγ (9), inhibitor of DNA binding 2, and (RUNX3) transcription factors (TFs) (8). Mature AMs can be distinguished by the expression of CD45, F4/80, CD11c, and SIGLECF markers. The downregulation and loss of CD11b expression occur during the maturation from fetal monocyte to the fully differentiated AM stage (10). The TFs that specify AMs compared with other tissue-resident macrophage populations have been described (11). Among these TFs, the early growth response 2 (EGR2) was proposed as an evolutionarily conserved, specific, mature-stage marker transcriptional regulator (11–13). However, the EGR2-mediated epigenetic mechanism and direct role in transcriptional regulation in AMs in normal homeostasis and under inflammatory conditions are still unclear.

Here, we characterized the contribution of myeloid EGR2 by analyzing alteration in chromatin openness, epigenomic marks, and the transcriptome, along with immune phenotyping and ex vivo and in vivo functional assays. Our work sheds light on the regulatory mechanisms; the proximal, likely direct target gene network controlled by EGR2; the effector molecules; and the pathways and finally the functional importance of those in response to pathogens and inflammation in AMs.

## Results

### Changing chromatin accessibility reveals a likely direct role for DNA-bound EGR2 in epigenomic regulation of AMs.

**Figure 1:**
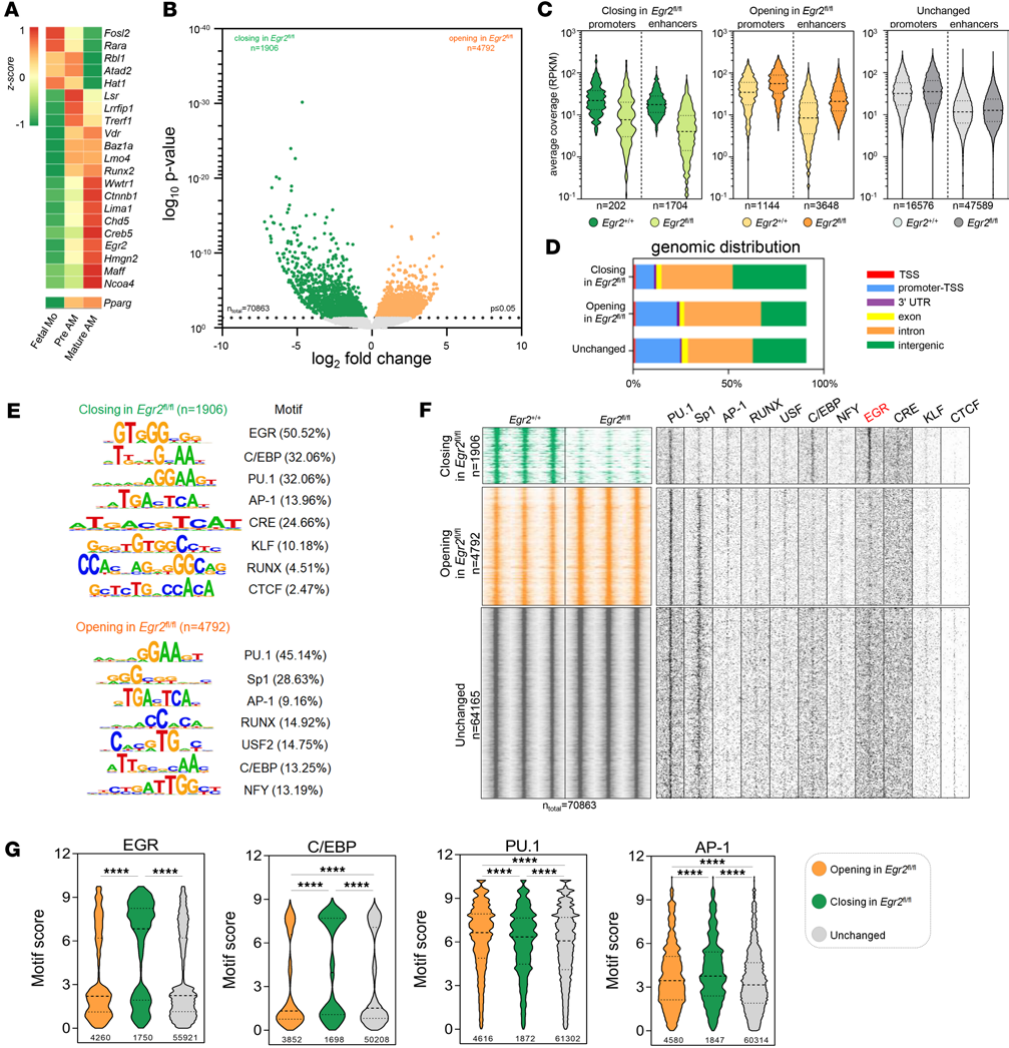
Changing chromatin accessibility reveals the role of EGR2 in epigenomic regulation of AMs. (**A**) Heatmap representation of single-cell-derived (ref. 37; Cohen et al., 2018; GSE119228) average gene expression values (*z* score) of AM-specific TFs and Pparg (ref. 11; Gautier et al., 2012) in fetal monocytes (Fetal Mo), pre-AMs (Pre AM), and mature AMs (Mature AM). (**B**) The volcano plot depicts the differentially opening (*n* = 4,792), closing (*n* = 1,906), and unchanged (*n* = 64,165) chromatin regions between *Egr2*^+/+^ and *Egr2*^fl/fl^ AMs determined by ATAC-Seq. (**C**) The violin plots visualize the average coverage (reads per kilobase of transcript, per million mapped reads; RPKM) values of the closing (green) and opening (yellow) DARs and unchanged (gray) chromatin regions between *Egr2*^+/+^ versus *Egr2*^fl/fl^ AMs (paired *t* test, *P* ≤ 0.05) at the promoter and enhancer regions. (**D**) The stacked bar chart represents the genomic distribution of DARs and unchanged chromatin regions between *Egr2*^+/+^ versus *Egr2*^fl/fl^ AMs (TSS, transcription start site; 3′ UTR, 3′ untranslated region; promoter-TSS, both TSS –1,000 bp/+100 bp and 5′ UTRs; intergenic, both intergenic and noncoding regions). (**E**) De novo motif enrichment of differentially closing (green) and opening (orange) chromatin regions for *Egr2*^+/+^ versus *Egr2*^fl/fl^ AMs. The significantly enriched motif matrices are presented along with the *P* value rank in the analysis, and the target% values are indicated in parentheses. (**F**) Read distribution heatmap shows the chromatin openness of the closing and opening DARs and unchanged chromatin regions in *Egr2*^+/+^ and *Egr2*^fl/fl^ AMs (2 kbp frames relative to the summit). Motif distribution heatmaps show the presence of indicated TF binding motifs (2 kbp frame relative to the summit). (**G**) Box plots show the motif scores of EGR, C/EBP, PU.1, and AP-1 motifs calculated on the opened DARs and unchanged chromatin regions (paired *t* test, *P* ≤ 0.05).

EGR2 is a TF, and we verified that it is expressed in mature AMs and at a much lower level in fetal AMs and pre-AMs (Figure 1A, Supplemental Figure 1A, and Supplemental Table 1; supplemental material available online with this article; https://doi.org/10.1172/jci.insight.164009DS1). In addition, it appears to be the dominant member of the EGR family in this cell type (Supplemental Figure 1B). This intriguing and specific expression pattern suggested a role for EGR2 in mature AMs, and thus we sought to examine its contribution to cell type specification and mechanistically link it to epigenomic and gene expression regulation. First, we generated an EGR2 myeloid-specific knockout (KO) mouse line by using the Lysozyme-Cre (LysCre) line crossed with an *Egr2*^fl/fl^ line (14) on the C57BL/6 background (referred to here as *Egr2*^+/+^ for the wild-type (WT) control and *Egr2*^fl/fl^ as the KO genotype). The modification of the *Egr2* locus leads to the deletion of exon 2 and results in total loss of EGR2 protein (14). In this model, we validated that the genetic elimination of EGR2 led to a close-to-complete reduction of mRNA level in both *Egr2* exons (approximately 65% decrease in exon 1 and 98% in exon 2) (Supplemental Figure 1C), and this resulted in undetectable EGR2 protein level by Western blotting (Supplemental Figure 1D).

We analyzed the different cell populations in the lung of control and EGR2-deficient mice using high dimensional flow cytometry applying a staining panel containing CD45, CD31, CD326, CD140a, CD3, CD4, CD8, CD45R-B220, TCR-γδ, CD11c, CD11b, CD64, CD103, MHCII, CD24, Ly6G, SiglecF, and Ly6C markers (Supplemental Figure 2A). The number of nonimmune subpopulations’ cells was similar, such as the CD31^+^ endothelial cells, the CD326^+^ epithelial cells, and the CD140a^+^ fibroblasts (Supplemental Figure 2B). In the case of immune cells we measured significant reduction in the number of mature AMs (CD11b^–^, CD11c^+^, CD64^+^), mature interstitial macrophages (CD11b^–^, CD11c^–^, CD64^+^), Ly6C^+^ monocytes, NK cells, B cells, and CD4^+^ and CD8^+^ T cells and elevation in the number of DCs. The other analyzed cell populations (Ly6C^–^ monocytes, neutrophils, eosinophils, γδ T cells) were essentially identical (Supplemental Figure 2C). These results show that the nonimmune cells are not affected by the EGR2 modification, but it has significant direct or indirect effect in the case of myeloid and lymphoid cells.

Next we more thoroughly analyzed the lungs of control and *Egr2*^fl/fl^ mice, and we found that myeloid-specific EGR2 deficiency led to a higher ratio of AMs based on H&E-stained and myeloperoxidase-labeled total lung histological samples (Supplemental Figure 3A) and defined by flow cytometry analysis using CD11b, F4/80, and CD11c markers (9) (Supplemental Figure 3, B and C) within the total immune cell population (CD45^+^ cells). The mature AMs showed low expression of CD11b and high expression of F4/80, CD11c, and SIGLECF marker proteins (15). As the result of EGR2 deficiency, we measured elevation in CD11b expression, reduction in CD11c expression, and near-total loss of SIGLECF expression based on flow cytometric measurements of AMs isolated from total lung homogenate and also from bronchoalveolar lavage (BAL) (Supplemental Figure 3, D and E). This analysis verified the expression pattern of canonical markers as recently described by others (12).

To complement the characterization of the immune phenotype, we analyzed the metabolic properties of these cells by obtaining AMs with BAL and ex vivo determination of mitochondrial oxygen flux and extracellular acidification rate (ECAR) using Agilent Seahorse XF Analyzer–based experiments. We obtained essentially identical oxygen consumption rate values in basal, etomoxir-dependent, and oligomycin-dependent respiration (Supplemental Figure 3, F and G) in *Egr2*^+/+^ and *Egr2*^fl/fl^ AMs. In contrast, the ECAR showed some reduction in *Egr2*^fl/fl^ cells (Supplemental Figure 3, H and I). Based on these data, we concluded that oxidative phosphorylation is intact, and the glycolytic flux is slightly reduced in EGR2-deficient AMs. We further concluded that myeloid-specific loss of EGR2 leads to a metabolically active macrophage that localizes in the alveolar space in higher numbers and shows a unique expressional profile of cell surface markers.

Next, we sought to understand the molecular details of EGR2’s mechanism of action in AMs, so we performed assay for transposase-accessible chromatin using sequencing (ATAC-Seq) and defined the differences in chromatin accessibility in EGR2-sufficient and -deficient cells. The 3 replicates highly correlated with each other in both conditions (*r* < 0.85) (Supplemental Figure 4A). Then we examined the differences in chromatin openness between control and KO cells. We found 1,906 closing and 4,792 opening chromatin regions with significant changes in *Egr2*^fl/fl^ AMs compared with control cells (Figure 1B and Supplemental Table 2). Both classes of differentially accessible regions (DARs) were localized in a higher proportion at enhancer than promoter regions, and the average coverage values were lower at enhancers (Figure 1C). The DARs were categorized by genomic distribution (Figure 1D). While the opening DARs and the unchanged regions showed a similar pattern with a relatively high representation of the promoter-transcription start site (TSS) (23.8% in DARs and 25.8% in unchanged regions), the number of the closing DARs was reduced in the promoter-TSS category (10.6%) (TSS –1,000 bp/+100 bp). In contrast, the closing DARs showed a higher representation of intergenic regions (42.5%) than the unchanged and opening DARs (26.2% and 31%). Importantly, this localization is characteristic of TF-bound regulatory enhancers (3). To determine the sequence motifs of direct DNA binding TFs under DARs, we applied de novo motif enrichment analysis. In the case of closed DARs, we detected a dominant enrichment of EGR (50.53%) followed by C/EBP (32.06%) motifs, and the binding motifs of general macrophage-specific lineage-determining transcription factors (LDTFs) such as PU.1, activator protein 1 (AP-1), and RUNX (Figure 1, E and F). By measuring the specificity of EGR, C/EBP, PU.1, and AP-1 motif sequences, it was outlined that the closing regions showed a large proportion of strong, canonical EGR and C/EBP motifs, while the strength of PU.1 and AP-1 motifs did not show any preference between the unchanged and DARs, which is in line with the proposed role of LDTFs (16) (Figure 1G). Remarkably, no enrichment of EGR motifs was detectable in the opening sites, suggesting that EGR2 is unlikely to act as a direct repressor via direct DNA binding.

Regarding the *Egr2* locus itself, our previous work in bone marrow–derived macrophages (BMDMs) described numerous regulatory enhancers (E1–24) based on RNA polymerase II chromatin immunoprecipitation sequencing suggesting autoregulation (13). Now, we compared the chromatin openness of AMs on these enhancer regions and identified 16 overlapping opened sites with the described enhancers and 10 AM-specific open regions (Supplemental Figure 4, B and C). We mapped the EGR binding motif on the *Egr2* locus and found 10 open sites containing it, which raises the possibility of EGR2 autoregulation in AMs. By measuring the enhancer RNA (eRNA) level as a surrogate of enhancer activity (17, 18) of 3 overlapping (E12, E13, and E23) and 3 AM-specific (E14.2, E24.1, E24.2) open sites containing EGR motifs, we detected a clear tendency to lower expression but no significant reduction in the case of overlapping enhancers, while the AM-specific ones were unaltered in EGR2-deficient AMs (Supplemental Figure 4D). These findings, along with a reduced exon 1 expression on its promoter (Supplemental Figure 1C), are compatible with EGR2 autoregulation; however, further studies are needed to conclusively establish it.

Based on these results, we concluded that EGR2 is a direct DNA binding TF in AMs, acting as an epigenomic factor required for chromatin openness, likely to activate gene expression at sites marked by macrophage LDTFs directly, and possibly regulating its own expression. Next, we decided to test EGR2’s molecular function by combining chromatin accessibility with transcriptomic analyses.

### EGR2 is a direct regulator of transcription and a key regulator of pathogen elimination–associated gene network.

**Figure 2:**
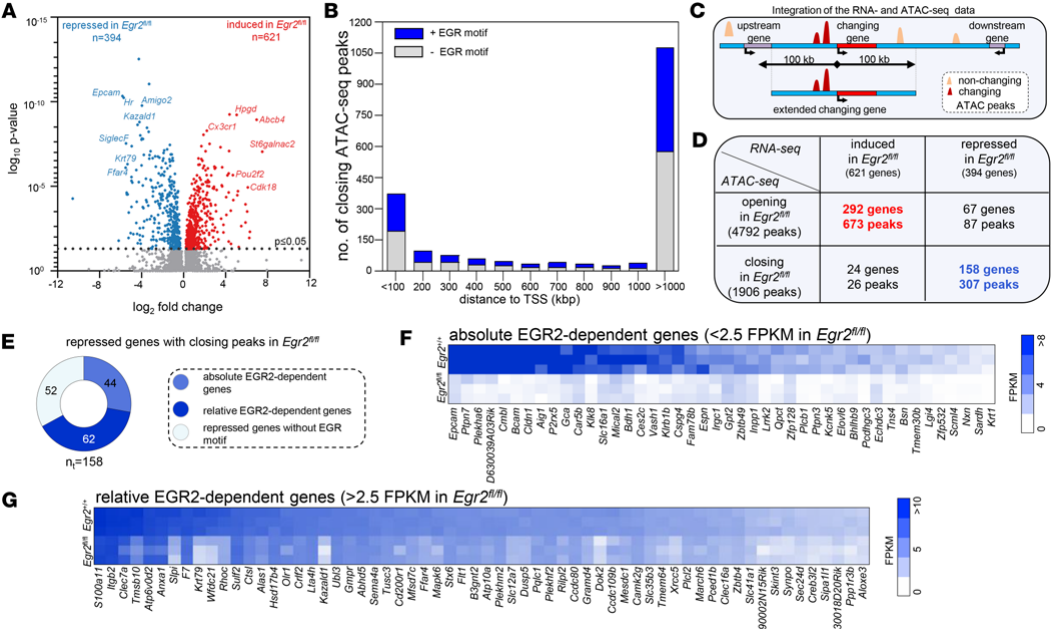
EGR2 is a late-stage regulator of AM gene expression establishing identity. (**A**) The volcano plot depicts the differentially expressed genes (DEGs) between *Egr2*^+/+^ and *Egr2*^fl/fl^ AMs. (**B**) The bar chart represents the distance (kbp) distribution of the EGR motif–containing (blue) or –noncontaining (gray) closing DARs relative to the repressed DEGs’ TSSs. (**C**) Schematic representation of the integration of DARs and DEGs. (**D**) The numbers of the DEGs and the associated DARs. (**E**) The pie chart depicts the ratio of the downregulated DEGs with closing DARs containing (moderate blue: absolutely EGR2-dependent, dark blue: relatively EGR2-dependent) or not containing EGR motif (light blue) in *Egr2*^fl/fl^ AMs. (**F** and **G**) Heatmaps represent the gene expression patterns of the repressed genes in *Egr2*^fl/fl^ AMs associated with EGR motif–containing closing DAR(s), separated by their expression, as lower (**F**) or higher (**G**) than 2.5 FPKM in *Egr2*^fl/fl^ samples.

To identify the molecular details of these proximal, likely directly activating transcriptional events, we assessed the relationship between potential EGR2-bound enhancers and the regulated genes. Therefore, we carried out bulk RNA-sequencing (RNA-Seq) experiments using control and *Egr2*^fl/fl^ AMs in the steady state and identified 364 significantly repressed and 621 significantly induced genes in EGR2-deficient AMs (*P* ≤ 0.05) (Figure 2A and Supplemental Tables 3–5). The mature AM markers *SiglecF*, *Krt79*, and *Kazald1* showed a robust decrease among the changing genes, while the *Cx3cr1* and *Abcd4* genes represented the most elevated ones (Figure 2A). By associating the closing DARs with the repressed DEGs, we found a high proportion of closed DARs within a 100 kbp distance (*n* = 373, from which 178 could be characterized by the presence of at least 1 EGR motif), but more than 1,000 DARs were localized at more than 1,000 kbp distance from a repressed gene, which suggests a chromatin and/or epigenomic remodeling effect of EGR2 (Figure 2B). Based on this conjecture, to further characterize the likely direct transcriptional regulatory role of EGR2, we integrated the DARs and DEGs by applying a stricter ±100 kbp distance/proximity from the gene’s TSS criterion, and we adjusted (shortened) the extended integrative region when a gene coding region was closer (Figure 2C). We defined 158 repressed DEGs as likely direct EGR2 targets, which were associated with 307 closed DARs based on the applied rule (Figure 2D and Supplemental Table 6). The EGR motif–containing closing DARs associated with repressed DEGs reveal that EGR2 is likely to act as a transcriptional activator in AMs. We then classified these proximal, likely direct target genes into repressed (*n* = 52) and absolutely (*n* = 44) and relatively (*n* = 62) EGR2-dependent ones (Figure 2, E–G, and Supplemental Table 7) based on the presence of the EGR motif and their gene expression levels. The gene expression baseline was set as 2.5 fragments per kilobase of transcripts per million mapped reads (FPKM) value in the *Egr2*^fl/fl^ condition. The genes with FPKM values below this threshold were considered nonexpressed ones. It should be noted that the absolutely EGR2-dependent genes are expressed at a lower level than the relatively EGR2-dependent ones (Figure 2, F and G).

**Figure 3:**
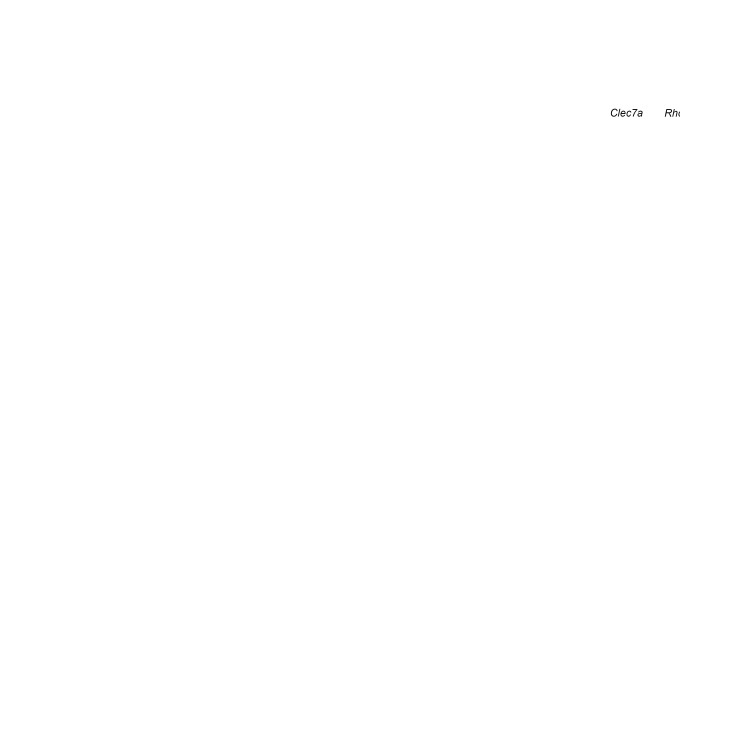
EGR2 affects gene expression and epigenomic features of AMs and embryonic stem cell–derived progenitors. (**A**) The box plots show the coverage (decile normalized average RPKM) of the H3K4me3 mark at the TSSs, BRD4 mark at EGR motif–containing coupled enhancers of absolutely and relatively EGR2-dependent repressed genes, repressed genes associated with closing DARs without EGR motif, and induced genes associated with opening DARs. (**B**) Integrative Genomic Viewer (IGV) snapshots represent the H3K4me3 mark at the indicated genes’ promoters and BRD4 signal at the coupled EGR motif–containing enhancers. (**C**) The box plots show the coverage (decile normalized average RPKM) values calculated from BRD4 CUT&RUN on the peak 200 bp regions of the EGR2-dependent peaks. (**D**) IGV visualization of RNA-Seq and ATAC-Seq coverages (overlaid) and the EGR motif on *Kazald1*. (**E**) The columns represent the relative eRNA expression (mean ± SEM) of *Kazald1*-related closing DARs with EGR motif in *Egr2*^+/+^ (*n* = 4) and *Egr2*^fl/fl^ (*n* = 5) samples. (**F**) The scheme of the gain-of-function experimental procedure applying ESC differentiation model toward myeloid progenitors. (**G**) The bar diagrams represent the relative mRNA expression (mean ± SEM) values of *Egr2* and AM marker *Cd11c* and *SiglecF* genes in untreated (-dox) or doxycycline-treated (+dox) myeloid progenitors (*n* = 4). (**H**) The box plots show the CD11c and SIGLECF proteins’ (median fluorescence intensity [MFI] ± SEM) in untreated (-dox) or doxycycline-treated (+dox) myeloid progenitors determined by flow cytometry (*n* = 4). (**I** and **J**) Column diagram representations of the relative mRNA (**I**) and eRNA (**J**) expression (mean ± SEM) values of representative EGR2-dependent genes (**I**) and the associated enhancers (**J**) in untreated (-dox) or doxycycline-treated (+dox) myeloid progenitors (*n* = 4) (*t* test, *P ≤ 0.05, **P ≤ 0.01, ***P ≤ 0.001, ****P ≤ 0.0001).

In order to obtain more direct evidence of the epigenetic regulatory role of EGR2 and to further characterize the epigenomic differences between WT and EGR2-null cells, we carried out CUT&RUN experiments (19) for the histone H3 trimethylated at lysine 4 (H3K4me3) mark to identify active gene promoters (20) and bromodomain containing 4 (BRD4) as a canonical active enhancer and promoter mark (21, 22). By measuring the H3K4me3 signal distribution on the downstream 500 bp region relative to the TSSs of absolutely and relatively EGR2-dependent genes’ TSSs, we found that the H3K4me3 levels correlated with the differences in gene expression values (Figure 3, A and B), further verifying that the primary effect of EGR2 is activation on chromatin structure and gene regulation. As a control, we also measured the H3K4me3 signal on the 292 induced gene TSSs (that could be associated with opening regions), which showed the expected opposite result (Figure 3A and Supplemental Table 8). In the case of BRD4, we obtained a similar pattern in promoters and enhancers which further strengthen the evidence regarding EGR2’s important epigenetic regulatory role (Figure 3, A–C). Moreover, we designed an eRNA-specific real-time quantitative PCR (RT-qPCR) assay for the DAR containing the EGR motif associated with the *Kazald1* gene to obtain direct evidence of the enhancer activity as a function of the presence of EGR2. We detected the eRNA production and the decrease of its level in EGR2-null AMs matching the reduction of the ATAC-Seq peak (Figure 3, D and E). Encouraged by these results, we performed gain-of-function experiments transfecting mouse embryonic stem cell–derived (ESC-derived) myeloid progenitors with a doxycycline-inducible EGR2 genetic construct (Figure 3F) (23). In this system, we were able to verify the overexpression of *Egr2* mRNA upon doxycycline treatment with RT-qPCR measurement (Figure 3G). Upon EGR2 overexpression, we were able to detect the significantly elevated level of CD11c and SIGLECF AM marker genes’ mRNA and protein (Figure 3, G and H). Then, we evaluated the transcriptional changes of selected candidate direct genes (*Kazald1*, *Clec7a*, *Atp6v0d2*, *Rhoc*) and the coupled enhancers. The analyzed representative genes and enhancers showed upregulated tendency following EGR2 overexpression (Figure 3, I and J). Importantly, based on these experiments, we concluded that the identified DARs act as enhancers, and their activity is EGR2 dependent.

**Figure 4:**
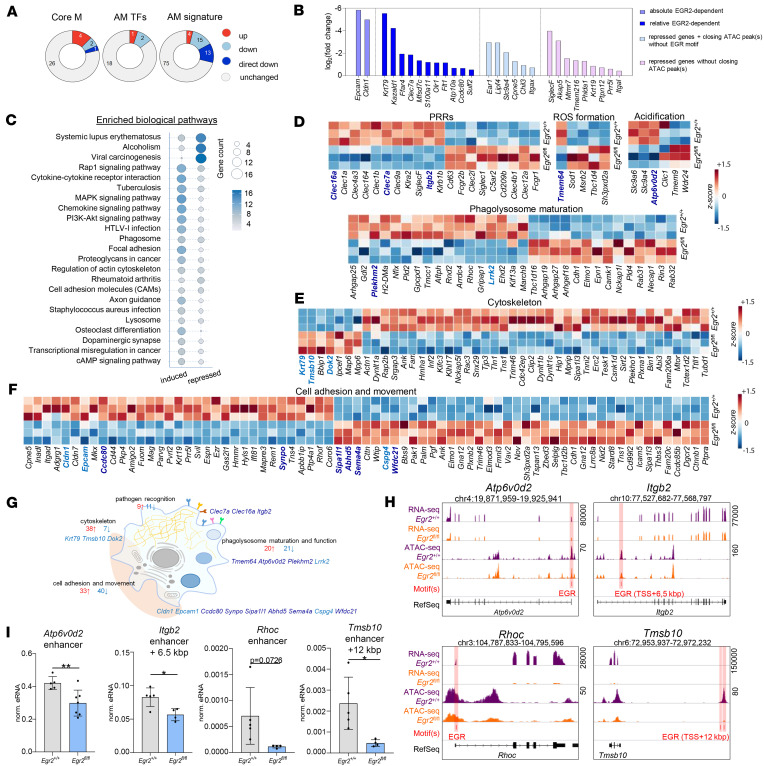
EGR2 deficiency affects gene networks linked to pathogen recognition and elimination. (**A**) Pie charts show the number of unchanged (gray), upregulated (red), downregulated (light blue), or directly downregulated (dark blue; associated with EGR motif–containing peaks) genes in *Egr2*^fl/fl^ samples that overlap with the core macrophage (Core M), AM-specific TF (AM TFs), and AM signature gene set (AM signature). (**B**) The log_2_(fold-change) values of downregulated AM signature DEGs on a column plot separated into 4 groups based on closing DAR association and the presence of EGR motif. (**C**) The proportional dot plot depicts the enriched KEGG biological pathways related to the 394 repressed and 621 induced genes in *Egr2*^fl/fl^ (RNA-Seq). The top pathways were selected based on the total number of the target genes and were depicted in that order. (**D**–**F**) The row-normalized heatmaps represent the significant expressional changes of phagocytosis-associated genes between *Egr2*^+/+^ and *Egr2*^fl/fl^ AMs for the (**D**) cytoskeleton organization and function (**E**) and cell adhesion and movement (**F**) pathways. The likely directly EGR2-regulated genes are highlighted in blue. (**G**) The number of the identified significantly up- and downregulated genes and the list of the relatively (dark blue) and absolutely (light blue) EGR2-dependent targets are depicted within the schematic representation of the cellular functions influenced by EGR2. (**H**) IGV visualization of RNA- and ATAC-Seq coverages and the presence of EGR motifs (highlighted in red) on *Egr2*^+/+^ and *Egr2*^fl/fl^ AMs on 4 selected likely direct EGR2 target genes (overlaid). (**I**) The columns represent the relative eRNA expression (mean ± SEM) values of selected repressed EGR2-dependent and phagocytosis-related genes’ associated closing DARs with EGR motif in *Egr2*^+/+^ (*n* = 5) and *Egr2*^fl/fl^ (*n* = 4) samples (*t* test, *P ≤ 0.05, **P ≤ 0.01).

Next, we examined the potential directly and indirectly EGR2-dependent DEGs from a functional point of view. The mRNA levels of core macrophage marker genes and AM signature genes (11) were partially altered in the absence of EGR2 (Figure 4A and Supplemental Table 9), but nearly half of the changed AM signature genes appear to be proximal EGR2 targets, such as *Epcam*, *Krt79*, *Cldn1*, and *Kazald1*, which showed log_2_ fold-change values between –4 and –6 (Figure 2I). We point out the high fold-change of *SiglecF* gene within the repressed group, which is the most canonical phenotypic marker of mature AMs (Figure 4B), and we demonstrated the near-total reduction of it at the protein level also by flow cytometric measurement (Supplemental Figure 3, D and E); based on the gain-of-function studies we demonstrated the EGR2-dependent induction of this protein (Figure 3, G and H).

We went on to predict the impacted biological pathways using the Kyoto Encyclopedia of Genes and Genomes (KEGG) database (24) applied to the list of DEGs. The top categories were related to pathogen elimination, sorted by the number of DEGs that overlapped with each pathway (Figure 4C and Supplemental Table 10). Using these pathways, we clustered the genes by their main biological functions. The transcriptional alterations linked to the absence of EGR2 are connected to pathogen recognition, ROS formation, phagolysosome acidification, and maturation. Moreover, these DEGs affect cytoskeleton organization, cell adhesion, and movement (Figure 4, D–G). Among the genes, some belong to a transcriptional network regulating pathogen elimination. Thus, we found key proximal targets of EGR2 such as the important proton pump–coding *Atp6v0d2* (25), the cell adhesive molecule–coding *Itgb2* (26), the cytoskeleton organizer small GTPase–coding *Rhoc* (27), and the β-thymosin–coding *Tmsb10*, which fulfill a function in cell motility (28) (Figure 4H). Again, using eRNA measurements, we could demonstrate the enhancers containing EGR2 binding sites and linked these to genes that have reduced activity in the absence of EGR2 (Figure 4I). Moreover, we detected reduced H3K4me3 signals at the promoter region and lower BRD4 signals at associated enhancers of *Kazald1*, *Atp6v0d2*, and *Rhoc* genes (Figure 3B). These data allowed us to conclude that EGR2 is a proximal, likely direct transcriptional regulator of terminal AM identity, phagocytosis, and pathogen inactivation.

### Lack of EGR2 leads to impaired zymosan phagocytosis.

**Figure 5:**
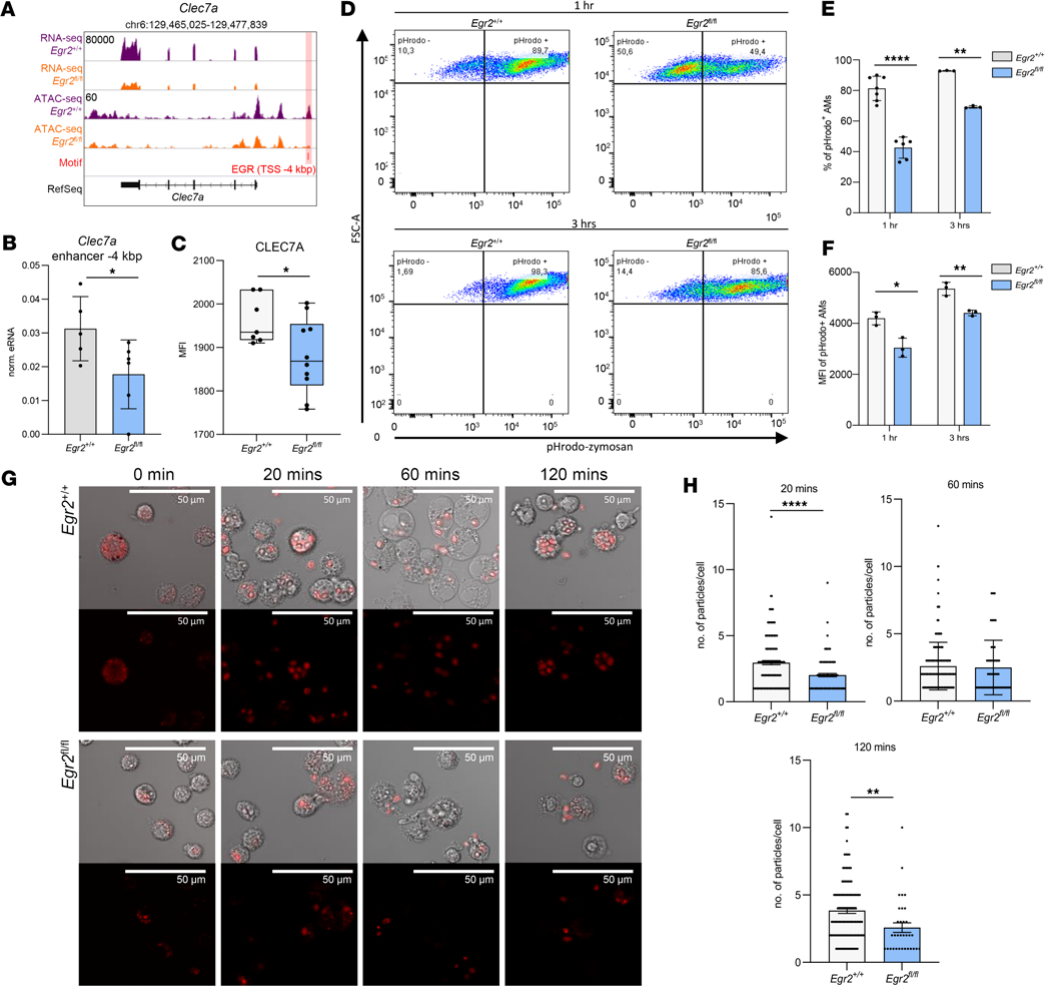
Lack of EGR2 leads to reduced Dectin-1 (CLEC7A) mRNA and protein expression and impaired zymosan phagocytosis in vitro. (**A**) IGV visualization of RNA-Seq and ATAC-Seq coverages of *Egr2*^+/+^ and *Egr2*^fl/fl^ AMs and the presence of EGR motifs (highlighted in red) on the *Clec7a* locus. (**B**) The columns represent the relative eRNA expression (mean ± SEM) values of *Clec7a*-related closing DARs with EGR motif in *Egr2*^+/+^ (*n* = 5) and *Egr2*^fl/fl^ (*n* = 5) samples. (**C**) The box plot shows CLEC7A (MFI ± SEM) expression of AMs isolated by BAL from *Egr2*^+/+^ (*n* = 7) and *Egr2*^fl/fl^ (*n* = 10) measured by flow cytometry. (**D**) Representative pseudocolor dot plots of *Egr2*^+/+^ and *Egr2*^fl/fl^ AMs after 1- or 3-hour pHrodo Red–conjugated zymosan treatment (flow cytometry). (**E**) Bar graphs represent the (mean ± SEM) percentage of nonphagocytotic (pHrodo^–^) and phagocytotic (pHrodo^+^) AMs after 1- and 3-hour pHrodo Red–conjugated zymosan treatment in *Egr2*^+/+^ (*n* = 7/*n* = 3) and *Egr2*^fl/fl^ (*n* = 6/*n* = 3) AMs (flow cytometry). (**F**) The mean ± SEM MFI values of phagocytotic (pHrodo^+^) AMs after 1- and 3-hour pHrodo Red–conjugated zymosan treatment in *Egr2*^+/+^ (*n* = 3) and *Egr2*^fl/fl^ (*n* = 3) AMs (flow cytometry). (**G**) Representative confocal microscopic images of *Egr2*^+/+^ or *Egr2*^fl/fl^ AMs 0, 20, 60, and 120 minutes after Texas Red–conjugated zymosan treatment. (**H**) The number of internalized Texas Red–conjugated zymosan bioparticles per 1 *Egr2*^+/+^ or *Egr2*^fl/fl^ AM upon 20, 60, and 120 minutes calculated from confocal microscopic images (*t* test, *P ≤ 0.05, **P ≤ 0.01, ****P ≤ 0.0001).

Our findings prompted us to examine some of the identified critical pathways in detail in light of the fact that we identified the *Clec7a* gene as a proximal target of EGR2 (Figure 5A). The BRD4 marks (Figure 3B) and eRNA levels (Figure 5B) of an associated enhancer were significantly lower in EGR2-deficient AMs on the *Clec7a*-associated enhancer, and the reduced H3K4me3 signal on the gene promoter (Figure 3B) further supports this notion. *Clec7a* is a gene coding for the Dectin-1 receptor, a canonical PRR of fungal cell wall components such as zymosan that plays a pivotal role in the antifungal response of AMs (29). We measured the expression of the Dectin-1 protein on the surface of control and EGR2-null AMs by flow cytometry of isolates from BAL. Like its mRNA, protein levels of Dectin-1 were significantly decreased in *Egr2*^fl/fl^ AMs (Figure 5C). As a functional readout for the activity of this protein, we analyzed the zymosan uptake of *Egr2*^+/+^ and *Egr2*^fl/fl^ AMs ex vivo using pHrodo-conjugated zymosan assay. The pHrodo dye gives a pH-dependent fluorescent signal in the acidic surroundings of the phagolysosome (30). We detected a significant decrease in the ratio of phagocytotic EGR2-null AMs versus WT AMs after 3 hours, and the capacity was approximately 70% (Figure 5, D and E). Moreover, we detected a 15% lower MFI of pHrodo signal in phagocytotic EGR2-deficient AMs (Figure 5F). In addition, we used confocal microscopy to follow the time course of zymosan uptake of AMs upon their ex vivo treatment with Texas Red–conjugated zymosan. We counted the number of internalized bioparticles at 0, 20, 60, and 120 minutes after treatment. The results showed a reduction in the average number of internalized zymosan bioparticles in *Egr2*^fl/fl^ compared with the control at the 20-minute time point. Even at the 120-minute point, the phagocytosis capacity of *Egr2*^fl/fl^ did not reach that of *Egr2*^+/+^; the quantity of the internalized zymosan in *Egr2*^fl/fl^ was lower (Figure 5, G and H). The impairment of Fc receptor–mediated phagocytosis was also measurable with the same method using pHrodo-conjugated *E*. *coli* bioparticles (Supplemental Figure 5, A and D), but the lack of EGR2 did not affect the uptake of *Staphylococcus aureus* bioparticles upon 3 hours of exposure and dextran macropinocytosis determined by pHrodo-conjugated assays (Supplemental Figure 5, B, C, E, and F). Thus, EGR2 deficiency leads to lower phagocytotic activity and internalization capacity of AMs especially upon zymosan stimulus, while the Fc receptor–mediated pathway is moderately affected, and the Scavenger receptor–mediated phagocytosis and macropinocytosis are not impacted.

### EGR2 influences the initial in situ transcriptional response to zymosan and leads to impaired resolution of inflammation.

**Figure 6:**
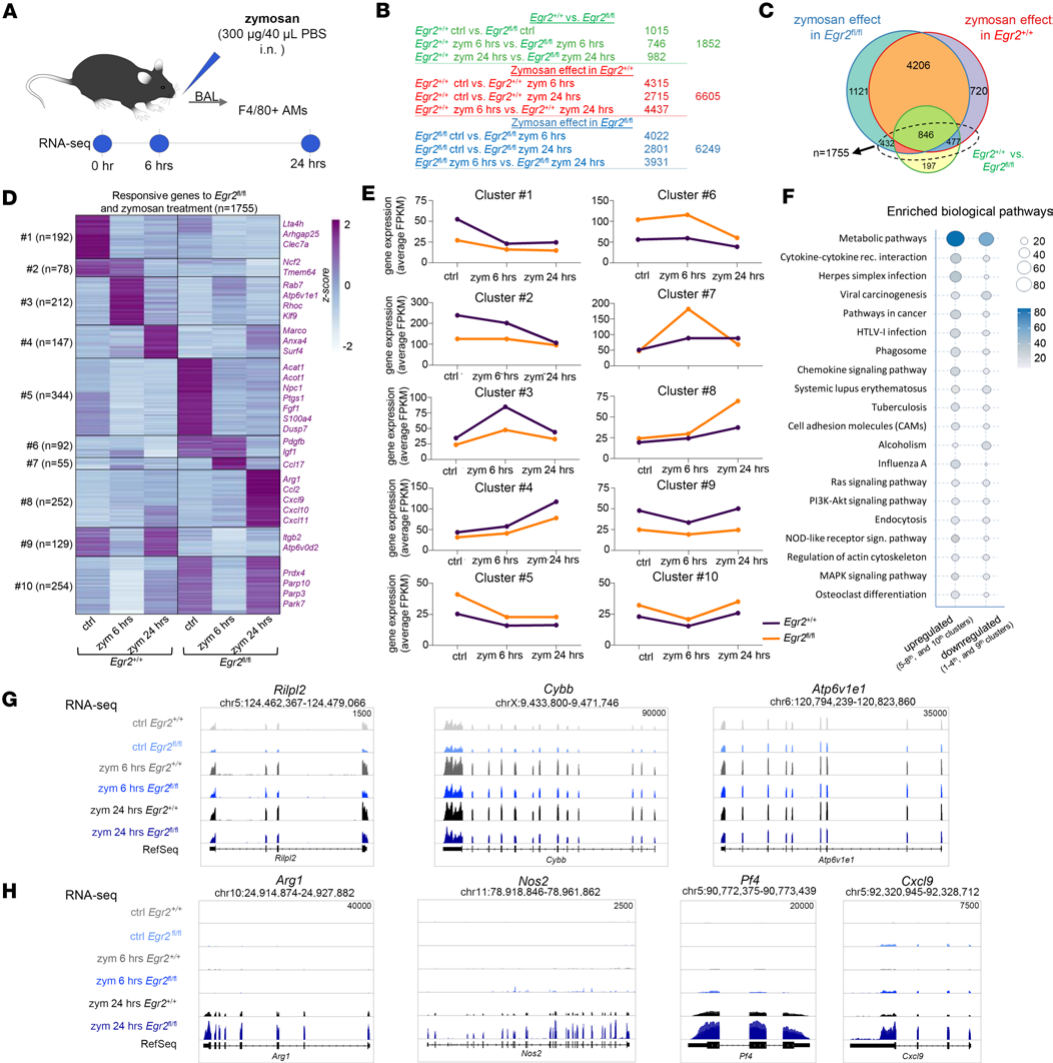
Lack of EGR2 partially and selectively impairs the zymosan-induced inflammatory response. (**A**) Schematic representation of sample preparation of in vivo zymosan. (**B**) The table contains the number of DEGs determined by RNA-Seq separately for the effect of EGR2 deletion (*Egr2*^fl/fl^) in each time point (green), for the effect of zymosan treatment in *Egr2*^+/+^ (red) and in *Egr2*^fl/fl^ (blue). The total values on the right indicate the number of individual changing genes in each comparison. (**C**) The Venn diagram represents the overlaps between the changing gene sets defined in panel **B**. The highlighted 1,755 genes represent a subset of genes that are responsive for both EGR2 deletion (*Egr2*^fl/fl^) and zymosan treatment. (**D**) K-means clustered (k_n_ = 10), row-normalized heatmap represents the average gene expression of 3 replicates of control and 6- and 24-hour zymosan treatments for the 1,755 genes in both *Egr2*^+/+^ and *Egr2*^fl/fl^ AMs. Representative genes of the clusters were listed on the right. (**E**) Line plots represent the average gene expression values of the clusters’ genes (depicted in panel **D**) in the control and 6- and 24-hour zymosan-treated *Egr2*^+/+^ (purple) and *Egr2*^fl/fl^ (orange) AMs. (**F**) The dot plot represents the top 20 KEGG biological pathways of these 1,755 genes. The pathways were selected and ordered based on the total number of the target genes. (**G** and **H**) IGV visualization of *Egr2*^+/+^ and *Egr2*^fl/fl^ RNA-Seq coverages in the control and upon 6- and 24-hour zymosan treatment on (**G**) selected phagocytosis-related (*Rilpl2*, *Cybb*, *Atp6v1e1*) and (**H**) selected inflammation-related (*Arg1*, *Nos2*, *Pf4*, *Cxcl9*) genes (overlaid).

Zymosan not only is phagocytosed but also induces a robust inflammatory response (31); therefore, we further characterized the early stage of zymosan-induced inflammatory response in vivo in *Egr2*^+/+^ and *Egr2*^fl/fl^ mice. We administered 300 μg zymosan intranasally and examined the induced changes after 6 and 24 hours (Figure 6A). The short time course ensured that the response of AMs was measured and no significant number of circulating monocyte-derived cells were present in the lung (12, 32). We determined the ratio and number of AMs and infiltrating polymorphonuclear cells (PMNs) by flow cytometry using CD45, F4/80, and Ly6G staining of BAL and did not find significant differences in the quantitative aspect of this cellular response (Supplemental Figure 6, A and B). Then we sorted the CD45^+^ and F4/80^+^ AM population from control and zymosan-treated samples. We performed bulk RNA-Seq from 4 biological replicates (Supplemental Figure 6C) and defined the zymosan-responsive and -unresponsive gene sets in *Egr2*^+/+^ versus *Egr2*^fl/fl^ AMs (Figure 6B and Supplemental Table 4). We identified 1,755 zymosan-responsive genes, which were altered in *Egr2*^fl/fl^ AMs compared with the control (Supplemental Table 11). The EGR2-dependent and zymosan-responsive gene set represented approximately 22.5% (*n* = 1,755) of the total zymosan-responsive genes (Figure 6C). It is important to note that the majority of the EGR2-independent zymosan-responsive genes (*n* = 6,047) contained numerous inflammatory TFs (e.g., *Stat3*, *Stat6*) and canonical effectors and cytokine-coding genes (e.g., *Il6*, *Il1a*, *Il1b*) showing dynamic expression changes, which were not altered by the lack of EGR2 (Supplemental Figure 6, F and G, and Supplemental Table 12), suggesting that EGR2 is not required for inflammatory gene expression per se and has a distinct and selective effect on early in situ inflammatory gene regulation.

**Figure 7:**
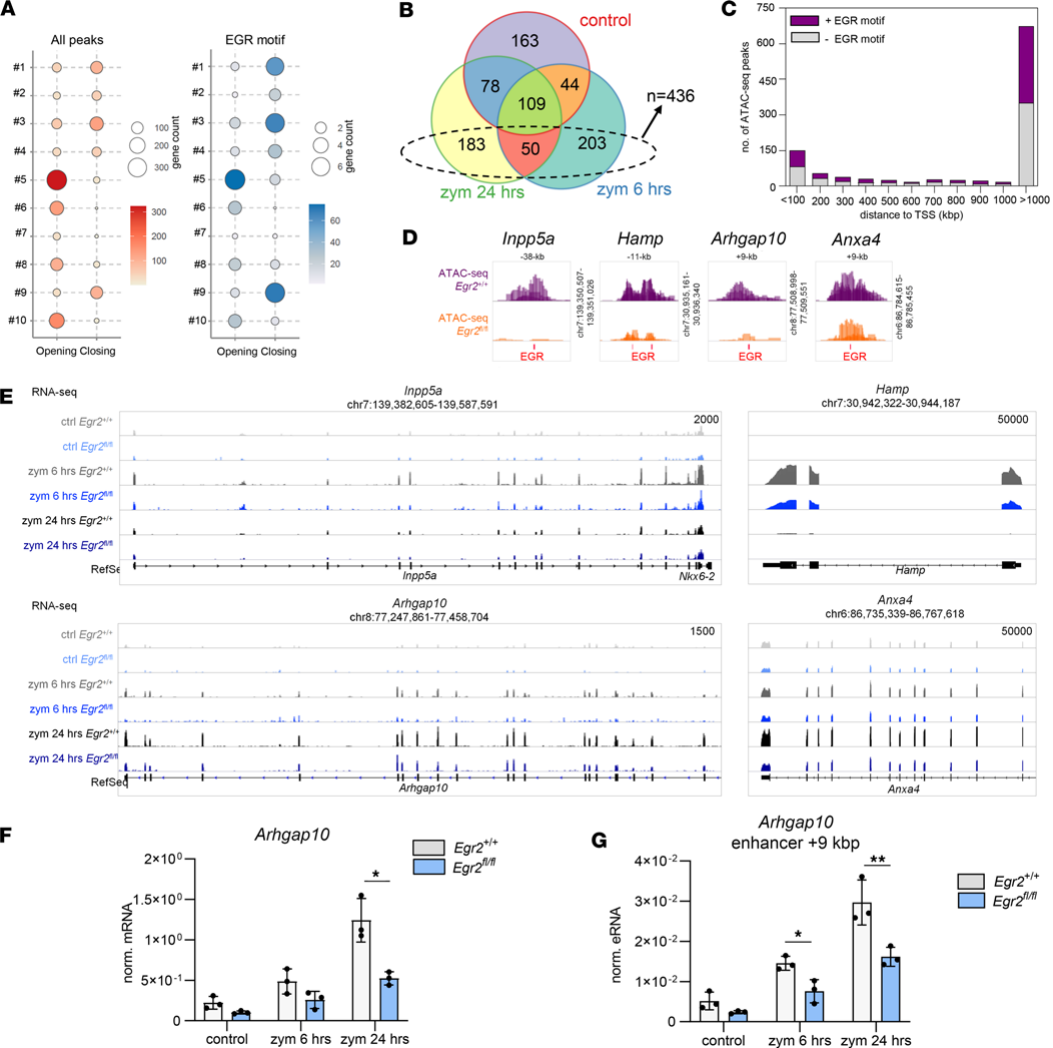
Lack of EGR2 affects some gene sets primarily regulated by zymosan stimulation. (**A**) The proportional dot plots represent the number of the associated opening and closing DARs within the ±100 kbp region of the gene TSSs or up to the nearest gene(s) (left, highlighted in red), and the number of the EGR motifs within these ATAC-Seq peaks (right, highlighted in blue), separately for the 10 clusters’ genes presented in Figure 6D. (**B**) The Venn diagram portrays the overlaps of the significantly repressed genes between *Egr2*^+/+^ and *Egr2*^fl/fl^ AMs upon control or 6-hour or 24-hour zymosan treatment. The highlighted 436 genes present a zymosan-dependent repressed gene set. (**C**) The bar chart represents the distance distribution of the EGR motif–containing (purple) or –noncontaining (gray) closing DARs (*n* = 1,075, which are located farther from the repressed genes than 1,000 kbp in Figure 2B) relative to the TSS position of the repressed zymosan-dependent DEGs (*n* = 436). (**D** and **E**) The IGV snapshots show (**D**) the ATAC-Seq coverages of *Egr2*^+/+^ and *Egr2*^fl/fl^ AM samples in control and in the presence of the EGR motif(s) related to 4 selected genes, (**E**) the coverages of which are shown in panel **D** in the control and 6- and 24-hour zymosan-treated *Egr2*^+/+^ and *Egr2*^fl/fl^ AMs in RNA-Seq (overlaid). (**F** and **G**) Bar graphs show the (mean ± SEM) values of normalized mRNA level of *Arhgap10* gene (**F**) and the *Arhgap10*-related region’s eRNA in the control and ex vivo 6- and 24-hour zymosan-treated *Egr2*^+/+^ and *Egr2*^fl/fl^ AMs (*t* test, *P ≤ 0.05, **P ≤ 0.01).

The 1,755 EGR2-dependent and zymosan-responsive genes were clustered into 10 groups based on their similarity in time-dependent gene expressional patterns (Figure 6, D and E) and were reintegrated with the previously defined closed DARs (*n* = 1,906) with the 10 EGR2-dependent and zymosan-responsive gene clusters. By applying the gene TSS extension as defined in Figure 2C, the genes of clusters 1 and 3 could be associated with the highest number of EGR motif–containing closing DARs, suggesting that these genes’ inflammatory regulation might be directly affected by EGR2 (Figure 7A). In addition, cluster 3 represents genes with the same basal expression and early induction.

We selected the zymosan-responsive and EGR2-dependent repressed DEGs, and we associated these genes with those closed DARs located more than 1,000 kbp distance from the TSS of DEGs in control conditions between *Egr2*^+/+^ and *Egr2*^fl/fl^ AMs (Figure 2B and Figure 7, B–D). We found new potential targets applying this method. Significantly, the expression of these genes (e.g., *Inpp5a*, *Hamp*, *Arhgap10*, and *Anxa4*) was influenced significantly by EGR2 during zymosan-induced inflammation but not under unstimulated, physiological conditions (Figure 7E). As a representative example, we validated ex vivo the mRNA expression profile of the *Arhgap10* gene and the eRNA level of associated DARs by RT-qPCR after 6- and 24-hour zymosan treatment. We measured a significant decrease in eRNA level at 6 hours and in both eRNA and mRNA levels upon 24-hour treatment in the *Egr2*^fl/fl^ condition (Figure 7, F and G). This observation suggests that EGR2 has a dual role in shaping the AM epigenome and alters various signal-dependent transcriptional responses without affecting the basal expression of the gene, thus acting as an epigenomic bookmarker.

**Figure 8:**
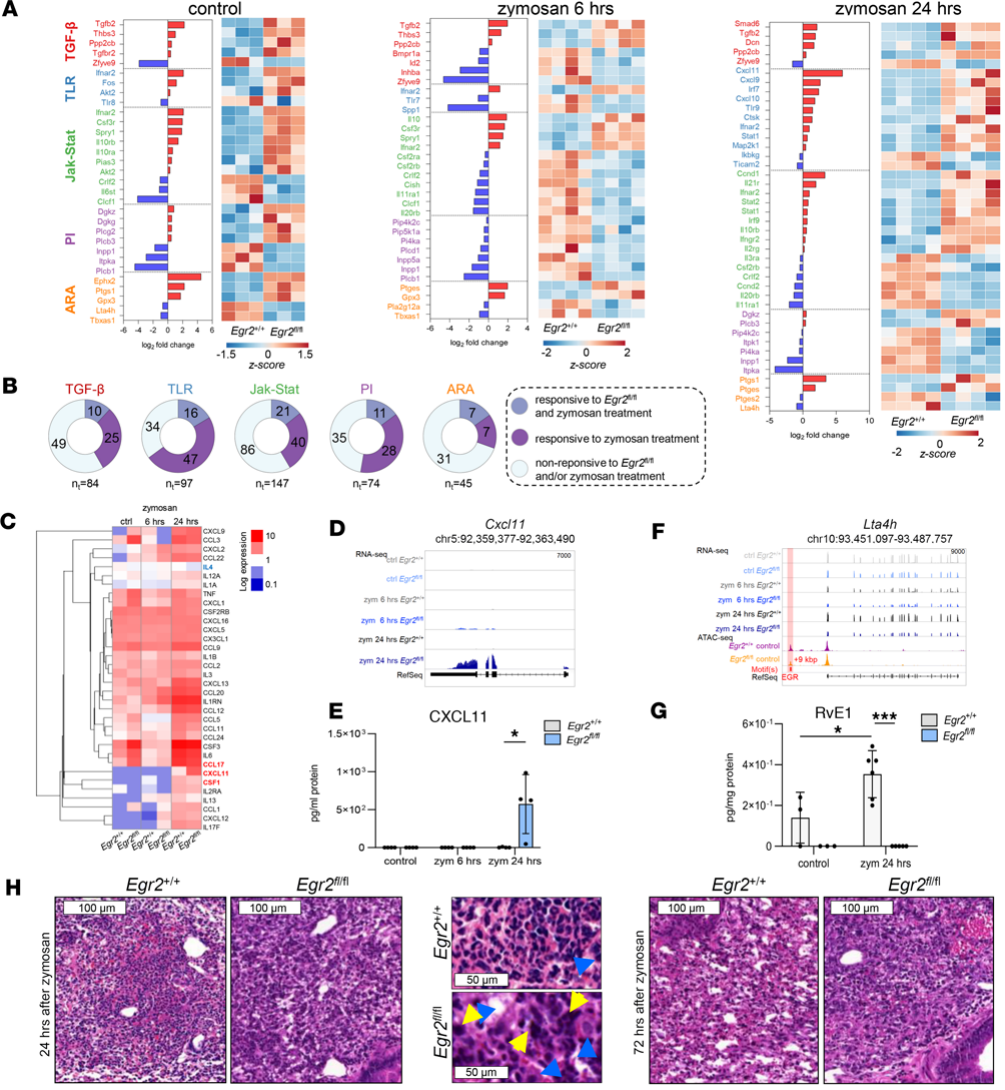
Characterization of the zymosan-mediated inflammatory gene expression and cytokine, chemokine, and lipid mediator production in vivo. (**A**) The bar charts and heatmaps represent the DEGs between the *Egr2*^+/+^ and *Egr2*^fl/fl^ AMs in control and upon 6- and 24-hour zymosan treatments. The selected signaling pathways (TGF-β, TLR, JAK/STAT, PI, and ARA) are depicted. The bar charts show the fold-differences in the average gene expression values, and the heatmaps show the row-normalized gene expression patterns of the depicted genes. (**B**) Pie charts show the number of genes from selected pathways that are nonchanging or changing significantly (*P* ≤ 0.05) upon different treatment conditions in *Egr2*^+/+^ and *Egr2*^fl/fl^ AMs (RNA-Seq). (**C**) The mean protein content of BAL fluid in control and 6- and 24-hour zymosan-treated samples determined by cytokine array (*n* = 4 in each treatment condition, 1-way ANOVA test with Tukey’s post hoc test, *P* ≤ 0.05). (**D**) IGV visualization of *Egr2*^+/+^ and *Egr2*^fl/fl^ RNA- and ATAC-Seq coverages. (**E**) The protein level of CXCL11 in BALF in different treatment conditions (cytokine array; *t* test, *P ≤ 0.05). (**F**) IGV visualization of RNA- and ATAC-Seq coverages of *Egr2*^+/+^ and *Egr2*^fl/fl^ AMs and the presence of EGR motifs on the *Ltah4* gene. (**G**) The amount of RvE1 in total lung homogenate of *Egr2*^+/+^ (*n* = 3) and *Egr2*^fl/fl^ (*n* = 6) mice analyzed by liquid chromatography with tandem mass spectrometry (LC-MS/MS) method (*t* test, *P ≤ 0.05). (**H**) Representative paraffin-embedded and H&E-stained lungs of *Egr2*^+/+^ and *Egr2*^fl/fl^ mice after 24- and 72-hour in vivo zymosan treatment. High magnification (original magnification, 630×) of H&E-stained sections shows that 24-hour after zymosan administration, there is a typical acute purulent inflammation with the presence of many PMNs in WT lungs. In *Egr2*^fl/fl^ lungs harboring EGR2-deficient AMs, besides PMNs, the inflammatory infiltrates contain an increased number of large macrophages (blue arrows), occasionally with large abnormal-appearing and chromatin-rich nuclei (yellow arrows).

From a functional aspect, the EGR2-dependent zymosan-responsive genes were related particularly to phagocytosis- and inflammation-related signaling pathways (Figure 6F). The IGV visualization of the expression profile of phagocytosis-related *Rilpl2*, *Cybb*, and *Atp6v1e1* genes and the induction of inflammatory markers *Arg1*, *Nos2*, *Pf4*, and *Cxcl9* demonstrated the robust influence of EGR2 in the regulation of these processes (Figure 6, G and H). We selected the KEGG gene sets of TLR, TGF-β, JAK/STAT, phosphatidyl-inositol (PI), and arachidonic acid (ARA) (27) signaling pathways to define the exact EGR2-dependent changes associated with the canonical inflammatory response. All pathways contained DEGs, but the affected genes showed variable ratios and gene expression patterns within the groups (Figure 8, A and B, and Supplemental Table 4). We observed mostly upregulated genes related to the TGF-β, TLR, and JAK/STAT pathways. The altered gene expression affected numerous cytokines (*Cxcl11*, *Cxcl9*, *Cxcl10*), receptors (e.g., *Tlr8*, *Tlr7*, *Tlr9*, *Csf3r*), and TFs (e.g., *Stat1*, *Stat2*, and *Irf9*). The DEGs associated with the PI signaling pathway were especially repressed and contained coding genes of phosphorylation cascade components (Figure 8A). Moreover, we identified the proximal, likely direct target *Lta4h*, which was consistently repressed upon zymosan treatment in the EGR2-null AMs (Figure 8, A and F). Because transcriptional alterations highly affected the level of several gene-coding cytokines and lipid mediator–synthesizing enzymes, we measured the cytokine profile by applying an ELISA-based cytokine array from BAL and the ARA metabolism-related lipid mediators by mass spectrometry from total lung homogenate. We found substantial downregulation of IL-4 at the protein level after 6-hour zymosan treatment and induction of CCL17, CXCL11, and CSF1 levels after 24-hour zymosan treatment. While the IL-4 is secreted by Th cells, the upregulated ones were specifically macrophage-derived cytokines (Figure 8, C–E). The induction of CXCL11 is particularly remarkable because this cytokine was not induced in the presence of EGR2 (Figure 8E), thus representing a de novo and very specific response. Among the ARA-derived lipid mediators, we detected significant basal level changes in the case of prostaglandin E2 and thromboxane B2 (Supplemental Figure 6H). In the control condition and following 24-hour zymosan administration, resolvin E1 (RvE1) was identified and increased with zymosan (Figure 8G). Remarkably, RvE1 was undetectable in the lungs of EGR2-deficient mice (Figure 8G). This lipid mediator is a product of leukotriene A4 hydrolase (LTA4H) enzyme and fulfills an important role in the resolution of inflammation (33). The expression level of *Lta4h* was reduced in zymosan-treated EGR2-null AMs (Figure 8F), though levels of leukotriene B_4_, the main product of LTA4H, were not altered in the lung by EGR2 deficiency at this time point (Supplemental Figure 6H). Again, it appears that EGR2 is a specific and selective regulator of a subset of distinct immune effector mechanisms in AMs.

We further characterized the inflammatory process by looking at the histology of paraffin-embedded, H&E-stained lungs upon 24- and 72-hour zymosan treatment. We observed comparable infiltration of PMNs in both WT and *Egr2*^fl/fl^ mice after 24 hours, though more macrophage-like mononuclear cells with large, irregular nuclei were present in *Egr2*^fl/fl^ lungs (Figure 8H). In addition, upon 72-hour treatment, the lung alveoli were unresolved in the lungs of *Egr2*^fl/fl^ mice, accompanied by shifting the tissue remodeling to incipient fibrosis (Figure 8H). Based on our results, EGR2 has a selective impact in the initial in situ transcriptional machinery of zymosan-induced inflammatory response, and the loss of this TF leads to an unresolved inflammatory phenotype in vivo via direct and indirect mechanisms.

### Insufficient response to Aspergillus fumigatus in EGR2-deficient AMs.

**Figure 9:**
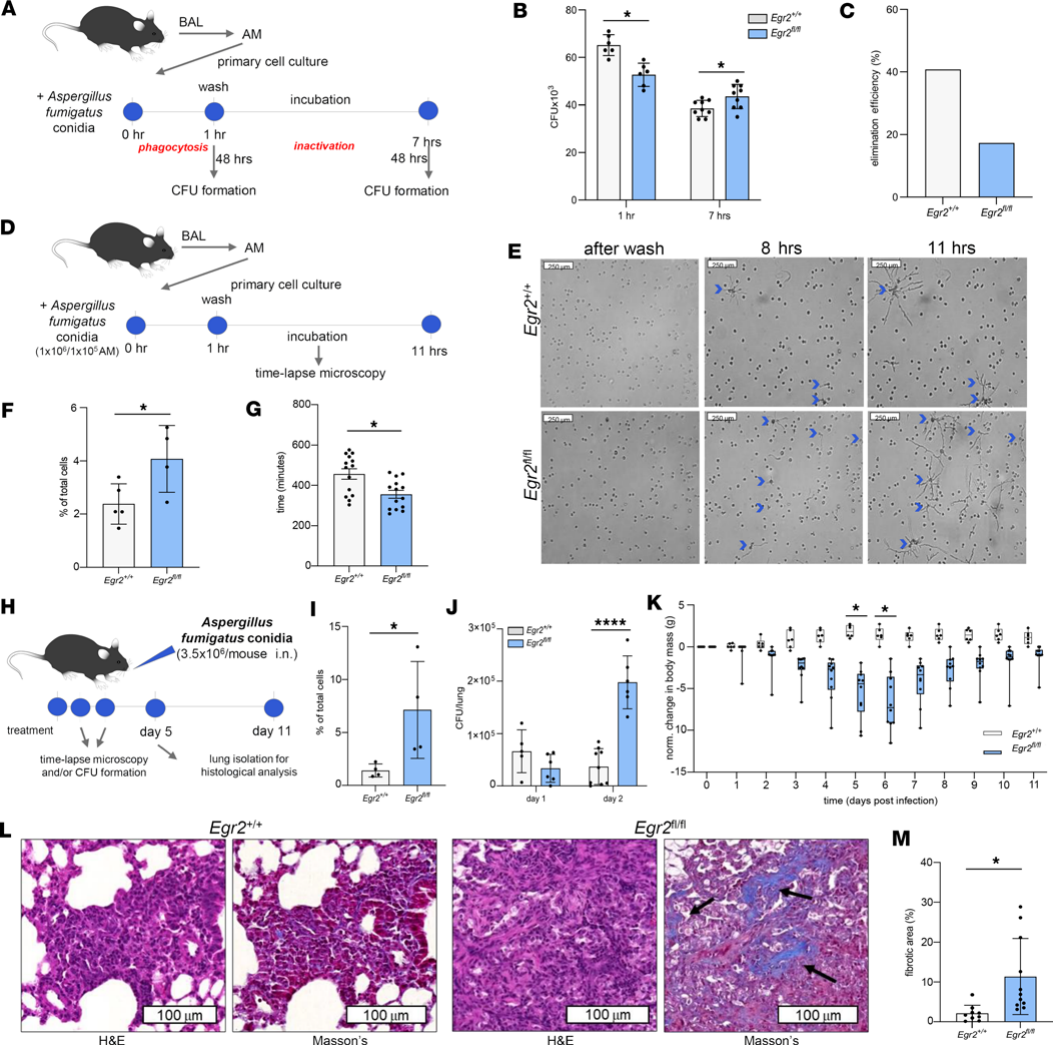
Impaired clearance of AF in myeloid EGR2-deficient mice. (**A** and **D**) Overview of the experimental setup to define the elimination efficiency of *Egr2*^+/+^ and *Egr2*^fl/fl^ AMs ex vivo by colony formation assay (**A**) and time-lapse microscopy (**D**). (**B**) The bar graphs represent the (mean ± SEM) values of AF colonies after 1 hour and 7 hours of cotreatment with *Egr2*^+/+^ (n_1hr_ = 6, n_3hrs_ = 9) and *Egr2*^fl/fl^ (n_1hr_ = 6, n_3hrs_ = 9) AMs. (**C**) The percentage of eliminated AF conidia based on the mean colony-forming capacity after uptake and elimination period in *Egr2*^+/+^ and *Egr2*^fl/fl^ AMs. (**E**) Representative images of time-lapse microscopy after washing the AF conidia from *Egr2*^+/+^ and *Egr2*^fl/fl^ AM coculture and at 8 and 11 hours (blue arrows: hypha-containing AMs). (**F**) The (mean ± SEM) value of hypha-containing AMs after 11 hours in *Egr2*^+/+^ and *Egr2*^fl/fl^ cells measured using time-lapse microscopic images (bar plot). (**G**) The (mean ± SEM) value of the start point of hypha growth in *Egr2*^+/+^ (*n* = 14) and *Egr2*^fl/fl^ (*n* = 14) AMs based on time-lapse microscopic images. (**H**) The schematic summary of in vivo AF infection model. (**I**) The (mean ± SEM) value of the percentage of hypha-containing *Egr2*^+/+^ and *Egr2*^fl/fl^ AMs after 24-hour AF infection based on time-lapse microscopic images (bar plot). (**J**) The bar plots represent the (mean ± SEM) values of AF colonies after 1- and 2-day AF infection in *Egr2*^+/+^ (n_day1_ = 5, n_day2_ = 9) and *Egr2*^fl/fl^ (n_day1_ = 6, n_day2_ = 6) lungs. (**K**) The change in body mass in *Egr2*^+/+^ (*n* = 6) and *Egr2*^fl/fl^ (*n* = 9) mice after AF infection normalized to the untreated mean total body mass. (**L**) Representative images of paraffin-embedded, H&E-stained and Masson’s trichrome–stained *Egr2*^+/+^ and *Egr2*^fl/fl^ lungs after 5-day AF infection (arrows: fibrotic area). (**M**) The percentage (mean ± SEM) of fibrotic area in inflamed fields based on Masson’s trichrome–stained lungs of *Egr2*^+/+^ (*n* = 9) and *Egr2*^fl/fl^ (*n* = 11) mice after 5-day AF infection (*t* test, *P ≤ 0.05, ****P ≤ 0.0001.

Finally, we extended our studies using pathophysiologically relevant fungal infection models. To compare the interaction of control and *Egr2*^fl/fl^ AMs, we used ex vivo and in vivo *Aspergillus fumigatus* (AF) infection studies (Figure 9, A, D, and H). First, we treated the *Egr2*^+/+^ and *Egr2*^fl/fl^ AMs isolated by BAL with AF conidia for 1 hour. During this period, the AMs could internalize the particles. At that point and after an additional 6-hour incubation, we lysed the cells and measured the colony-forming capacity of the samples (Figure 9A). We found a significant decrease in CFUs after 1-hour incubation and a significant increase after 7 hours in the case of EGR2-null AMs (Figure 9B). In addition, the ratio of internalized and inactivated AF conidia was lower in *Egr2*^fl/fl^ AMs (Figure 9C). Applying time-lapse microscopy, we followed the morphological changes after the internalization of AF conidia (Figure 9, D and E). We found that the EGR2 deficiency of AMs caused a higher ratio of hypha-containing AMs and an earlier start point of hypha growth compared with the WT cells (Figure 9, F and G). Next, we analyzed the in vivo effect of AF conidia using intranasal infection. To characterize the pulmonary fungal burden especially in the innate immune period, first we obtained BAL from *Egr2*^+/+^ and *Egr2*^fl/fl^ lungs, and we plated the CD45^+^ cells to cell culture dishes. We followed the changes with time-lapse microscopy. Applying this method, we detected a higher ratio of hypha-containing AMs in *Egr2*^fl/fl^ lungs (Figure 9I) compared with the WT. Furthermore, upon 2-day AF infection, we detected a substantially higher colony formation capacity of *Egr2*^fl/fl^ lung homogenates compared with *Egr2*^+/+^ ones (Figure 9J), establishing the role of myeloid EGR2 in antifungal response in vivo. We monitored the changes in body mass and performed histological analysis of the lungs (Figure 9, K–M). Clinically, we could not detect any significant differences upon infection in WT mice under the applied conditions. However, the *Egr2*^fl/fl^ mice rapidly started to lose body weight, likely indicating the presence of inflammation (Figure 9K). The largest differences were measurable during the fifth and sixth days of infection. Accordingly, the comparative histological reviews revealed a tendency of nonresolving protracted inflammation with early progressive pulmonary fibrosis in myeloid-specific EGR2-deficient lung samples at day 5 of AF inoculation, which was verified by Masson’s trichrome staining (Figure 9K and arrows, Figure 9L). It is remarkable that normal control lungs harboring WT AMs showed regressing inflammation at day 5 with partial alveolar resolution but without fibrosis (Figure 9M).

AF is an opportunistic pathogen, and severe or lethal AF infection exclusively affects patients who are immunocompromised. Therefore, cyclophosphamide-induced (CP-induced) immunosuppression is a suitable model, which leads to fulminant aspergilloma formation in the lung (34–36). We applied this agent combined with gentamicin bacterial prophylaxis to analyze the impact of myeloid-specific loss of EGR2 in immunosuppressive conditions (Supplemental Figure 7A). First, we measured the changes in the numbers of different immune cells (AMs, monocytes, PMNs, B cells, CD4^+^ and CD8^+^ T cells) upon 24-hour infection in the lung by flow cytometry. The usage of CP caused significant reductions in the numbers of B and CD4^+^ T cells and a slight, not significant, reduction tendency in the case of AMs and CD8^+^ T cells in the lung of control and EGR2-deficient mice compared with the nonimmunocompromised infection. The reduction of monocyte and neutrophil numbers was observable only in control animals (Supplemental Figure 7B). Next, we analyzed the BAL fluid isolated from the lungs upon 1 day of AF infection. We could not detect significant alteration in the total protein levels during different conditions applying the BCA method (Supplemental Figure 7C). Under immunosuppression, we measured significantly higher lactate dehydrogenase activity (Supplemental Figure 7D) and elevated cytokine concentration tendency of inflammatory (TNF-α, IL-6) and profibrotic (CXCL9, CXCL11, CXCL13) markers by ELISA in *Egr2*^fl/fl^ mice compared with the controls (Supplemental Figure 7, E and F). The survival rate was similar in control and *Egr2*^fl/fl^ mice (Supplemental Figure 8A), while the weight loss was significantly higher at day 10 after infection in the case of *Egr2*^fl/fl^ ones (Supplemental Figure 8B). Based on histological analysis of paraffin-embedded lungs stained by H&E, periodic acid–Schiff, and Grocott’s method, we found AF hyphae containing substantially enlarged aspergillomas (Supplemental Figure 8, C–E) and necrotizing inflammation, which is composed of predominantly large macrophage-like mononuclear cells with pleomorphic, large nuclei or apoptotic cells (Supplemental Figure 8F), in the *Egr2*^fl/fl^ animals. The *Egr2*^fl/fl^ lungs contained persisting inflammatory cells, and induced large spindle cells, corresponding to activated fibroblasts’ morphologies (Supplemental Figure 8F, yellow arrows). The Masson’s trichrome staining of such regions shows coarse collagen deposits reflecting significant fibrosis (Supplemental Figure 8F, blue fibrillar staining). Together, these results provide additional in vivo evidence regarding the importance of EGR2’s role in the antifungal activity of AMs while we must point out that the observed findings are the reflection of a complex multicellular phenotype.

## Discussion

Tissue-resident macrophages are specified by distinct sets of lineage-specific TFs, which reflect their different microenvironments and functional demands (11, 37). EGR2 is likely acting as such a TF in lung AMs (13, 37). However, its AM-specific role, mechanism(s) of action, and direct contribution to gene expression regulation are unknown. Here we showed the following. (i) EGR2 activates gene expression as a likely DNA-bound transcriptional activator. (ii) EGR2 is a proximal regulator of a small subset of late-stage lineage markers and many phagocytosis-related genes in AMs (20). (iii) EGR2 regulates Dectin-1 and Dectin-1–mediated phagocytosis of zymosan and fungi. (iv) A portion of the zymosan-induced inflammatory events are modulated by myeloid EGR2 as an epigenomic bookmarker, resulting in increased and/or de novo–induced profibrotic cytokines (i.e., CXCL11) and abolished production of resolving lipid mediators (i.e., RvE1) in EGR2 deficiency. (v) Myeloid EGR2 deficiency in AMs leads to impaired resolution of inflammation and defect in killing of fungi during AF infection.

### The role of EGR2 as an accessory or secondary LDTF.

AM identity is determined by general macrophage LDTFs such as PU.1 (7) and C/EBPβ (38) and by the lipid-regulated nuclear receptor, PPARγ, of which the latter has been shown to be required for AM differentiation (9). It appears, though, that EGR2 is a unique component of AMs in respect to its specialized function as it directly activates only a relatively small portion of mature AM marker genes (such as *SiglecF*), while several other markers remain unchanged (i.e., *Car4*, *Il1rn*, and *Krt19*). Therefore uniquely, the loss of EGR2 does not create a total differentiation blockade and a wide range of functional deficits like PU.1 in all macrophage types, as PPARγ does in AMs (9, 39). This assessment is supported by our data as EGR2 deficiency resulted in only a partial loss of differentiation-dependent gene expression (Figure 4, A and B), while largely intact inflammatory and phagocytic responses were observed (Figure 6C and Supplemental Figure 6). Therefore, EGR2 appears to be an accessory or secondary LDTF rather than a primary one by acting in concert to guide specification, complementing the role of other factors, and refining certain aspects of AM identity and function.

### The dual mechanism of EGR2 action.

EGR2 has been implicated in myeloid cell, monocyte, and macrophage differentiation, acting both as transcriptional activator and repressor (40, 41). However, some of the claims regarding a decisive role in controlling macrophage versus neutrophil differentiation were refuted using genetic models showing that neither in vitro nor in vivo deletion of a combination of EGRs impact macrophage differentiation per se (42). In addition, EGR2 has been shown to participate in defining the terminal stage of human monocyte to macrophage differentiation (43). Recent work from our group mechanistically positioned EGR2 as a linchpin between IL-4–mediated, STAT6-dependent alternative polarization and the mediation of long-term stable polarization (13), which Glass and colleagues (44) also demonstrated in murine BMDMs. Multiple reports identified EGR2 as a TF associated with AMs (8, 9, 11, 13), and recently a requirement for this factor has been established using a genetic model similar to ours (12). Building on all these pieces of information, we expanded our understanding by showing here that EGR2 is, with all likelihood, part of the AM epigenome. We could show that EGR2 is likely to be a DNA-bound transcriptional activator required for chromatin opening and gene activation in the steady-state unstimulated AMs. This could be deduced from the fact that the chromatin regions closing in the absence of EGR2 are highly enriched for the EGR motif (Figure 1E). Regions opening in the absence of EGR2 do not have enrichment for EGR2’s canonical binding site, and therefore it is unlikely that EGR2 acts as a repressor for a large set of genes as a DNA-bound factor. Therefore, the repressive effects are either via tethering to other proteins or indirect. Determination of the AM EGR2 cistrome will help clarify this proposal and the mechanism of it. Importantly, the other TF binding site enriched at the closing site is C/EBP, especially when compared with the rest of the changing (opening) and not changing sites (Figure 1F). This observation suggests the possibility of a collaborative interaction between EGR2 and C/EBP in AMs.

Our approach was based on the combination of defining chromatin openness and binding sites based on motif analyses coupled with eRNA expression validation as a surrogate of a particular enhancer’s activity in the proximity of a regulated gene. This allowed us to identify and call direct proximal targets and their enhancers and classify them as ones having an absolute or relative requirement for EGR2 based on expression levels and thresholds (Figure 2, E–G). These calls were validated by the CUT&RUN-based determination of active promoter histone mark H3K4me3 and showed a robust correlation. As far as the mechanistic aspects of gene activation are concerned, a coenrichment of C/EBP (Figure 1G) suggests that the 2 factors likely act collaboratively. This finding is in line with previous findings in human monocyte-derived macrophages, where EGR2 motifs were found to contribute to the action of the PU.1–AP-1–C/EBP regulator complex (43). Further studies are required to clarify the interrelationship and hierarchy between these factors and their upstream regulators (i.e., factors required for EGR2 induction). Autoregulation (13) (Supplemental Figure 4, B–D) could contribute to the maintenance of EGR2 expression.

Regarding the downstream events regulated, a key proximal target is Dectin-1 (Figure 5A). This gene appears to be directly regulated by EGR2 and, along with a set of genes (i.e., *Atp6v0d2*, *Itgb2*, *Rhoc*), contributes to phagocytosis, following zymosan induction, and to antifungal response. This finding places EGR2 directly upstream of this important and clinically relevant pathway (see below), which involves recognition, internalization, and killing of fungi mediated by Dectin-1, making it a specific coordinator of this set of pathways.

The response to zymosan as a prototypical response to pathogens is also skewed in the absence of myeloid-specific EGR2, revealing an additional role for this TF. Again, a large portion of the inflammatory response is intact (Supplemental Figure 5F), suggesting that most signaling pathways and mechanisms do not require or are altered by the presence of EGR2, while a distinct set of specific ones are impaired. Here 2 mechanisms can be invoked. One is the impact of genes controlled by EGR2 in the steady-state, unstimulated cells (i.e., *Epcam*, *Tmsb10*) and/or impact on genes requiring EGR2 only upon external stimuli, here zymosan, such as *Anxa4* and *Arhgap10*. This latter mechanism posits that chromatin-bound EGR2 is required for inflammatory gene expression without impacting the basal level of expression and setting the inducibility and/or amplitude of induced inflammatory gene expression. Some of these effects could be difficult to link to EGR2-bound enhancers, such as the de novo induction of CXCL11 (Figure 8E), and thus can be indirect. However, the reduced levels of eRNAs of *Kazald1*, *Clec7a*, *Atp6v0d2*, and *Rhoc* suggest a direct role of EGR2-bound enhancers. This mode of action suggests that EGR2 has a silent bookmarking function only revealed if the cell is exposed to distinct stimuli. The existence of such a mechanism would explain the large number (around 1,000) of closing genomic sites in EGR2-deficient cells having EGR2 motifs (Figure 1E) and the fact that we could not link these sites to genes modulated by the absence of EGR2 in the unstimulated macrophages. However, some of these sites bound by EGR2 would contribute to noncognate signaling events such as the zymosan response.

### Lung pathophysiology and the clinical relevance of the EGR2 deficiency.

Finally, the histological findings of the lungs at steady state and upon zymosan and AF treatment are consistent with the highlighted transcriptional and phenotypical changes. In steady state, the myeloid-specific deficiency of EGR2 leads to higher cellularity in interalveolar spaces, and we found some mononuclear cells with larger size and irregular nuclear structure, which may be a feature of poorly differentiated AMs (Supplemental Figure 3A). Such a morphological and cellular alteration in lungs with EGR2-deficient AMs was more obviously detected in pathogen-activated inflammatory cells of either AF or zymosan-treated pulmonary tissues (Figure 8H). It was especially apparent when observing the inflammatory infiltrate for cellular details and compared with zymosan-treated inflamed control lungs harboring WT AMs. Accordingly, the large macrophage-like inflammatory cells with prominent irregular blastoid nuclei are preferentially manifested in lungs with EGR2-deficient AMs only (Figure 8H). During zymosan- or AF-induced inflammations, we detected prolonged inflammations with higher fibrosis in the EGR2-KO lungs (Figure 8H and Figure 9J). The observed fibrotic patterns within the bronchiolar and peri-bronchiolar chronic active inflammatory lesions appear morphologically comparable with the human disease entity, called “bronchiolitis obliterans with organizing pneumonia” (45). Applying the CP-based immunosuppression model, which reflects the immunocompromised background of patients with invasive aspergillosis, we provided additional evidence for the greater aspergilloma formation capacity and enhanced fibrosis in the lung. An elegant set of studies by Perlman and colleagues including lineage tracing have shown that AMs are replaced by circulating monocytes upon injury over several weeks, and these monocyte-derived cells are responsible for fibrosis (15, 32, 46). However, circulating monocytes are unlikely to play a role in the studies we presented because (i) we analyzed the epigenomic and transcriptomic state of steady-state unstimulated AMs and (ii) the time course of the zymosan almost certainly (6- and 24-hour) and the AF treatment very likely (5-day) are much shorter to reasonably expect significant infiltration. Besides monocytes, neutrophils play an important role in the antifungal process. In the absence of further evidence, we should mention that the applied myeloid-specific EGR2 deficiency may cause phenotypic difference in PMNs, which potentially modulate the described complex pathological phenotype.

In summary, our work is substantially expanding prior studies and shows that EGR2 is a specialized epigenomic and positive transcriptional modulator of AM gene expression in the steady state and also during inflammatory gene expression employing at least 2 distinct mechanisms of action, direct transcriptional regulation and silent bookmarking to control cell type–specific gene expression and function. Given its restricted function in AMs, one can envision targeting it with small molecules or via its upstream regulators to control AM function and antifungal response.

## Methods

### Sex as a biological variable.

Sex was not considered as a biological variable. Both male and female 3-month-old mice were used.

### Mouse strains.

The mice were bred under specific pathogen–free conditions at the Laboratory Animal Core Facility of the University of Debrecen. The genetic background was C57BL/6. The *Egr2*^fl/fl^ mouse strain was a gift from the laboratory of Patrick Charnays (PSL University, Paris, France). We crossed the *Egr2*^fl/fl^ line with LysCre mice to reach the myeloid-specific KO condition (*Egr2*^fl/fl^). These animals were crossed back for 8 generations with C57BL/6J strain. As control, we applied *Egr2*^+/+^ LysCre littermates (*Egr2*^+/+^).

### Isolation of AMs by BAL.

Mice were euthanized by isoflurane inhalation. After preparation of the trachea, we applied 20G endotracheal canul (Kent Scientific) via washing the bronchoalveolar space with 3× 800 μL of PBS solution supplemented with 1% FBS and 200 mM EDTA pH 7.4 per mouse. The cells were centrifuged for 10 minutes at 800*g* and 4°C. We removed the fluid phase and resuspended the cells in 200 μL of ammonium-chloride-potassium lysis buffer for 2 minutes to lyse residual blood cells. Cells were then centrifuged with the same settings to pellet cells. We applied the isolated AMs and supernatant for further experiments (see below and in the Supplemental Methods) (RNA-Seq, ATAC-Seq, CUT&RUN, flow cytometry and cell sorting, mouse cytokine array, ELISA, and lipid mediator analysis).

### Phagocytosis assays.

The AMs were isolated by BAL and were purified with CD45 magnetic beads (Miltenyi Biotec). We cultured 100,000 cells in 6-well plates in 1 mL RPMI medium containing 5% penicillin, 5% streptomycin, and 10% FBS. After attachment, we added 1 × 10^6^ pHrodo Red–labeled zymosan (Invitrogen) for 1 replicate. Upon 60- and 120-minute incubation we scraped the cells and measured them by flow cytometry. We described the analysis of phagocytosis by confocal microscopy in the Supplemental Methods.

### Fungal strains and preparation of AF cultures.

The applied fungal strain was AF 293 cultured at 37°C on a nitrate minimal medium (47). To obtain conidia for infections, organisms were grown for 6 days. Conidia were harvested with PBS supplemented with 0.1% Tween 80 and concentrated by centrifugation (5,000*g* for 5 minutes at room temperature) (48). The applied AF treatment models and microscopic analysis are described in the Supplemental Methods.

### In vivo zymosan-induced lung inflammation.

Mice were anesthetized by isoflurane inhalation. A total of 300 μg zymosan (Invitrogen) was suspended in 40 μL PBS and administered intranasally. Upon 6- and 24-hour incubation the mice were euthanized by isoflurane. The lungs were harvested for further analysis, or the cellular components of BAL were isolated for flow cytometry and cell sorting.

### Ex vivo zymosan treatment of AMs.

The AMs were isolated by BAL and were purified with CD45 magnetic beads (Miltenyi Biotec). We cultured isolated cells in 24-well plates in 0.5 mL RPMI medium containing 5% penicillin, 5% streptomycin, and 10% FBS. After attachment, we added zymosan (50 μg/mL, Invitrogen). Upon 6- and 24-hour incubation total RNA was isolated by TRIzol (Invitrogen) for mRNA and eRNA transcripts’ detection (Supplemental Methods).

### Statistics.

Based on the RNA-Seq experiments the DEGs were determined with 1-way ANOVA supplemented with a post hoc Tukey’s honestly significant differences statistical test by using the stats, tidyverse, and sqldf packages in R. Considering all RNA-Seq samples (22 in total), genes that showed higher expression than 1 FPKM at least in 2 samples were considered expressed and were included in the comparative analyses in which *P* ≤ 0.05 significance cutoff was applied. We applied 1-way ANOVA test with Tukey’s post hoc test to determine the significance of protein content of BAL fluid measured by cytokine array. A 1-tailed *t* test was used in other experiments. Differences between sample groups were considered significant if *P* value was equal to or less than 0.05. Box plots in figures show the interquartile range, median (line), and minimum and maximum (whiskers). GraphPad Prism v.9 software was applied for statistical calculations.

### Study approval.

All animal experiments were carried out in the Laboratory Animal Core Facility of University of Debrecen under the supervision of the Animal Care Committee of the University of Debrecen (Debrecen, Hungary). The experimental protocol was approved by the Animal Care Committee (4/2017 DEMÁB, 14/2019/DEMÁB). Animal experiments conformed to the general guidelines of the European Community (86/609/EEC) and special guidelines of biosafety level 2 (200/54/EC).

### Data availability.

The generated next-generation sequencing datasets are available in the NCBI Gene Expression Omnibus repository under GSE181087 and the other datasets in the Supporting Data Values XLS file.

## Author contributions

ZK produced the RNA-Seq and cytokine microarray samples. ZK and PT carried out the ATAC-Seq experiments. WKB prepared the CUT&RUN and Western blots. SP contributed to library preparation and sequencing. DB and LH performed the computational analyses. TK, P Bai, and ZK carried out the Agilent Seahorse measurements. A Patsalos and MS contributed to targeted LC-MS/MS analysis of lipid mediators. BD performed the histological analysis. P Boto, KB, ZK, IS, AVR, and FRD performed the flow cytometric measurements. LF, GV, and ZK performed the confocal microscopic imaging of zymosan uptake. ZV and ZC contributed to ex vivo zymosan treatment of AMs and RT-qPCR experiments. VB, ZMS, and ZK carried out the AF-related experiments. A Pap contributed to mouse strain management. ZK and DB designed the figures. ZK, DB, and LN planned the project and drafted the manuscript. LN supervised the work. All authors discussed the results and commented on the manuscript. The order of co–first authorship was determined by weighing relative contributions to experimental design, execution, analyses, conceptualization, and writing of the manuscript.

## Supplementary Material

Supplemental data

Unedited blot and gel images

Supplemental table 1

Supplemental table 10

Supplemental table 11

Supplemental table 12

Supplemental table 13

Supplemental table 2

Supplemental table 3

Supplemental table 4

Supplemental table 5

Supplemental table 6

Supplemental table 7

Supplemental table 8

Supplemental table 9

## Version 1 07/23/2024

In-Press Preview

## Version 2 09/10/2024

Electronic publication
