# Supplementary material for: EGR2 is an epigenomic regulator of phagocytosis and antifungal immunity in alveolar macrophages
Source: JCI Insight. 2024 Sep 10;9(17):e164009. doi: 10.1172/jci.insight.164009 (PMC11385099; doi:10.1172/jci.insight.164009)
Supplement: Supplemental data [file jciinsight-9-164009-s048.pdf]

# Figure S1.

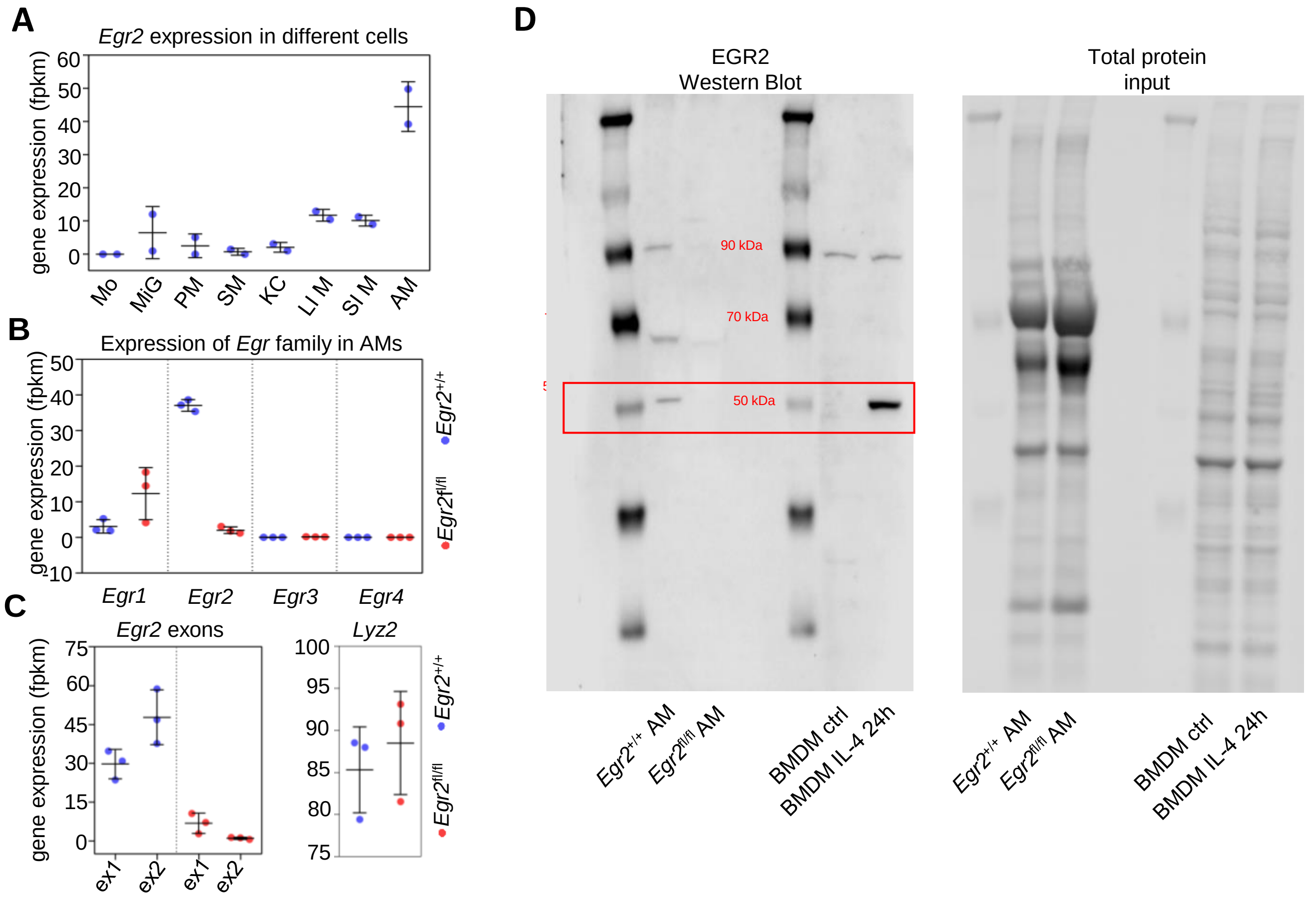


**Figure S1. Lack of EGR2 expression in alveolar macrophages of *Egr2*$^{fl/fl}$ mice.**
**(A-C)** Scatter plots show the gene expression of *Egr2* in different types of macrophages (source of data: RNA-seq, GSE63340, MiG: microglia, PM: peritoneal macrophage, SM: splenic macrophage, KC: Kuppfer cell, LI M: large intestinal macrophage, SI M: small intestinal macrophage, AM: alveolar macrophage) and monocytes (Mo) (data source: GSE63341) **(B)** the gene expression of the *Egr* gene family members, **(C)** the *Egr2* exons and the *Lyz2* gene in *Egr2*$^{+/+}$ and *Egr2*$^{fl/fl}$ AMs. **(D)** EGR2 Western Blot analysis of *Egr2*$^{+/+}$ and *Egr2*$^{fl/fl}$ AMs. Control and IL-4 treated BMDMs were applied as negative and positive controls.

**Figure S2.**

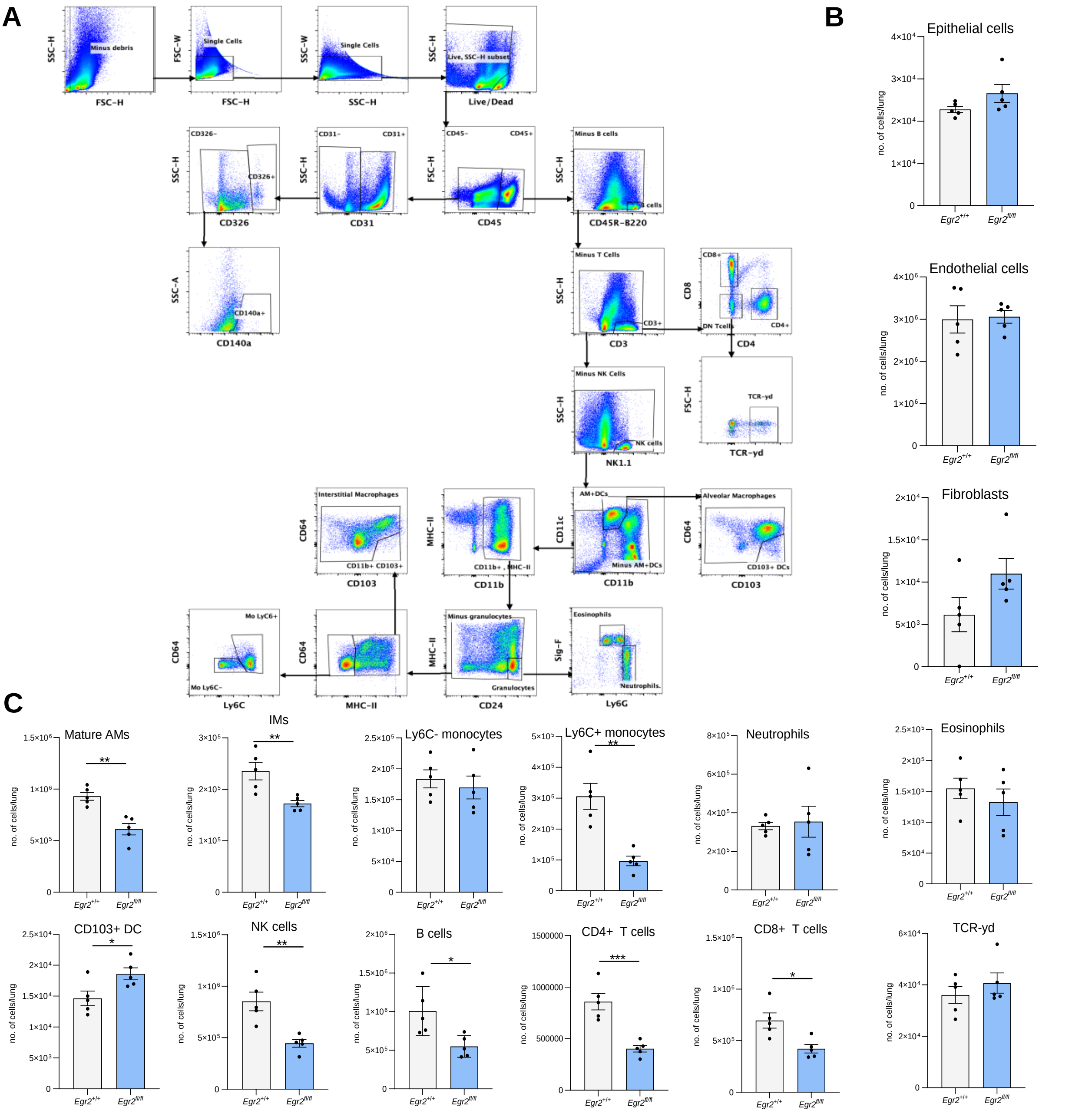


**Figure S2. Characterization of non-immune and immune cell populations in the lung of *Egr2*$^{fl/fl}$ mice.**
**(A)** The applied gating strategy for identify the different cell populations in mouse lung by flow cytometry. **(B-C)** The mean±SEM of number of non-immune **(B)** cells (epithelial cells, endothelial cells, and fibroblasts), and immune **(C)** cells (matured alveolar macrophages (AMs), interstitial macrophages (IMs), Ly6C$^{+}$ and Ly6C$^{-}$ monocytes, neutrophil granulocytes, eosinophil granulocytes, CD103+ dendritic cells (DCs), natural killer (NK) cells, B cells, CD4$^{+}$, CD8$^{+}$, and γδ T cells) of total lung samples from *Egr2*$^{+/+}$ and *Egr2*$^{fl/fl}$ (n=5) mice. (Student's t-test, p≤0.05).

# Figure S3.

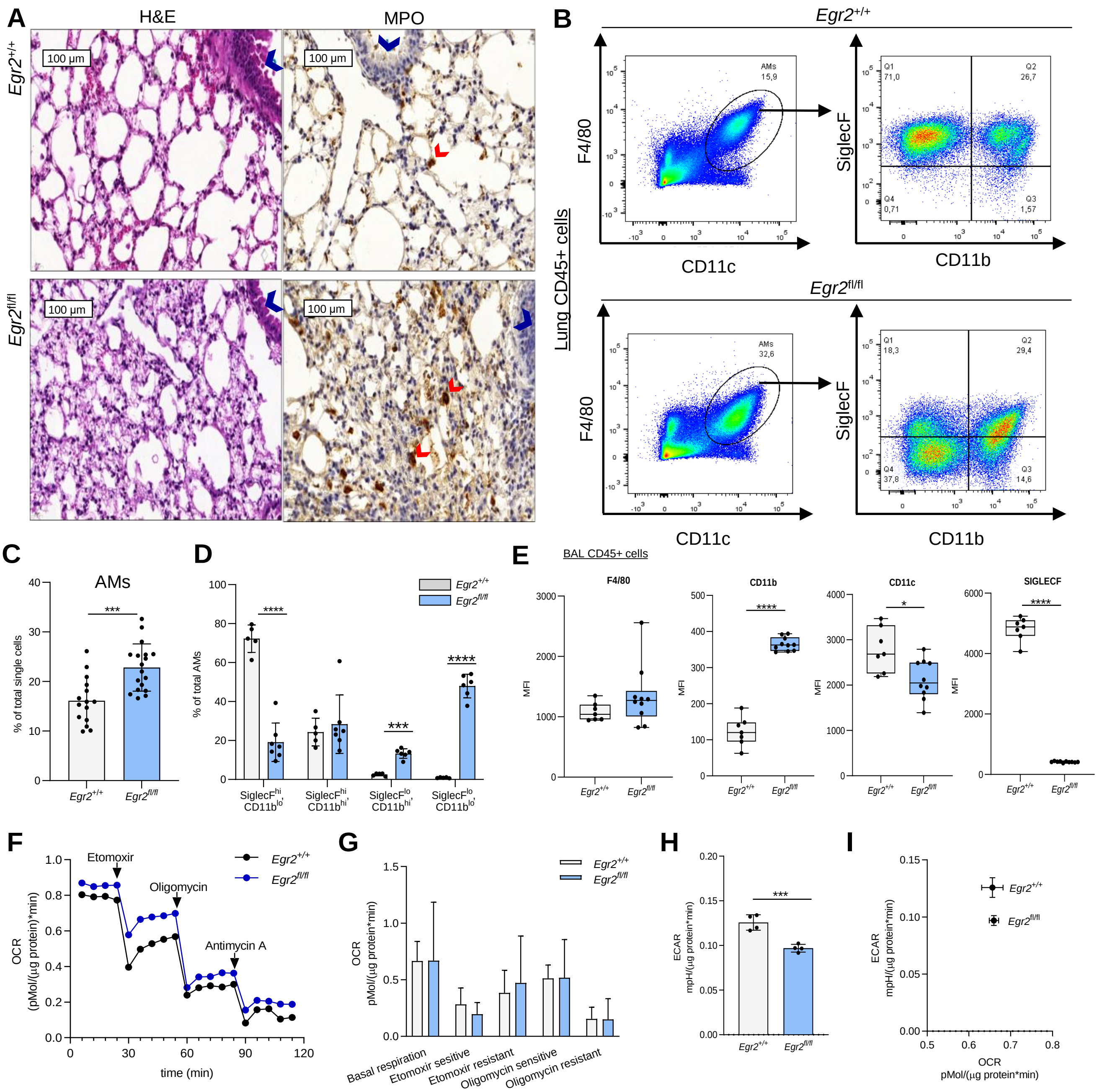


**Figure S3. Characterization of the alveolar macrophages of *Egr2*$^{fl/fl}$ mice.**

**(A)** Representative images of paraffin-embedded, H&E- and MPO-stained lung samples of *Egr2*$^{+/+}$ and *Egr2*$^{fl/fl}$ mice (blue arrows: bronchus, red arrows: MPO$^{+}$ cells). **(B)** Representative pseudo color dot plots of CD45$^{+}$ cells of the total lung of *Egr2*$^{+/+}$ and *Egr2*$^{fl/fl}$ mice marked by F4/80 and CD11c antibody. The double-positive population was gated and the expression of SiglecF and CD11b were measured (flow cytometry). **(C)** The mean±SEM percentage of F4/80$^{+}$ and CD11c$^{+}$ cells (AMs) within the CD45$^{+}$ population of total lung samples from *Egr2*$^{+/+}$ (n=15) and *Egr2*$^{fl/fl}$ (n=18) mice. **(D)** The mean±SEM ratio of different subpopulations of AMs based on SigelcF and CD11b expression in the lung of *Egr2*$^{+/+}$ (n=5) and *Egr2*$^{fl/fl}$ (n=7) mice. **(E)** The box plots show the MFI of AM markers in CD45$^{+}$ cells isolated from BAL and measured by flow cytometric method in *Egr2*$^{+/+}$ (n=5) and *Egr2*$^{fl/fl}$ (n=10) samples. **(F)** The mean±SEM OCR of *Egr2*$^{+/+}$ (n=7) and *Egr2*$^{fl/fl}$ (n=5) AMs isolated from BAL and measured by Agilent Seahorse Analyzer. **(G)** The bar graphs represent the mean±SEM OCR values after etomoxir, oligomycin, and antimycin A treatment in *Egr2*$^{+/+}$ (n=7) and *Egr2*$^{fl/fl}$ (n=5) AMs isolated from BAL and measured by Agilent Seahorse Analyzer. **(H)** The mean±SEM ECAR values of *Egr2*$^{+/+}$ (n=4) and *Egr2*$^{fl/fl}$ (n=4) AMs (Agilent Seahorse Analyzer measurement). **(I)** Mean OCR and ECAR values were plotted against each other. (Student's t-test, p≤0.05).

# Figure S4.

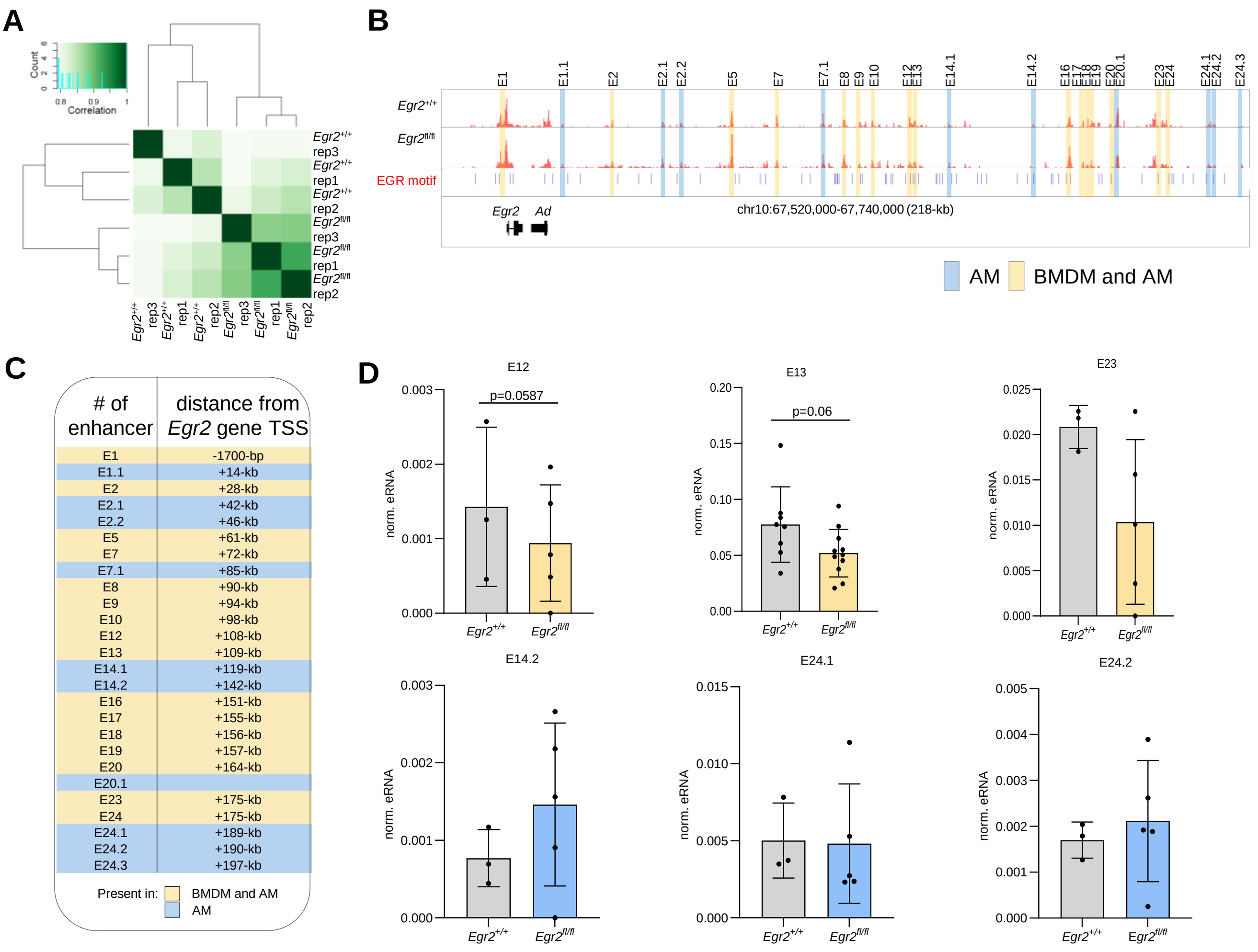


**Figure S4. The possibility of EGR2 autoregulation in alveolar macrophages.**
**(A)** The correlation heatmap represents the similarity among ATAC-seq replicates. **(B)** IGV snapshot of *Egr2* locus, the common enhancers (yellow) in AMs and BMDMs or AM-specific (blue) enhancers based on ATAC-seq and annotated EGR binding motifs (red). **(C)** Positions of enhancers in *Egr2* locus and their presence in BMDMs or AMs. **(D)** The mean±SEM values of normalized eRNA levels of EGR motif-containing common (E12, E13, and E20; yellow) and AM-specific (E14.2, E24.1, and E24.2; blue) enhancers in *Egr2*$^{+/+}$ (n=3) and *Egr2*$^{fl/fl}$ (n=5) AMs measured by RT-qPCR (Student's t-test, p≤0.05).

## Figure S5.

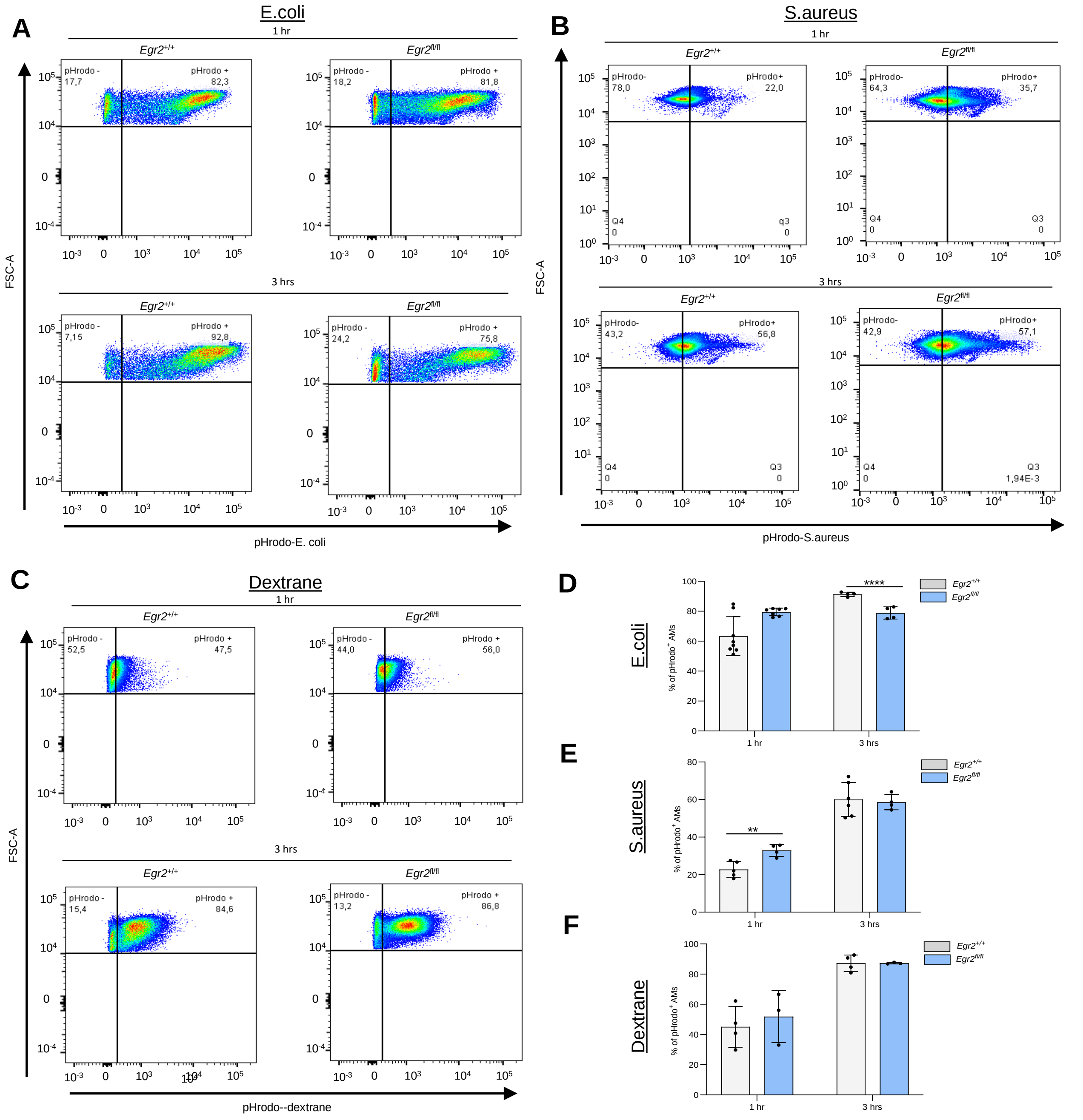


**Figure S5. Characterization of phagocytosis of alveolar macrophages of *Egr2*$^{fl/fl}$ mice.**
**(A, B, C)** Representative pseudo color dot plots of CD45$^{+}$ and F4/80$^{+}$ *Egr2*$^{+/+}$ and *Egr2*$^{fl/fl}$ AMs after 1 hr or 3 hrs pHrodo-Red conjugated E.coli **(A)**, S.aureus **(B)** or dextran **(C)** treatment (flow cytometry). **(D, E, F)** Bar graphs represent the mean±SEM percentage of non-phagocytotic (pHrodo$^{-}$) and phagocytotic (pHrodo$^{+}$) AMs after 1 hr and 3 hrs pHrodo-Red conjugated E.coli **(D)**, S.aureus **(E)** or dextran **(F)** treatment in *Egr2*$^{+/+}$ (n=4) and *Egr2*$^{fl/fl}$ (n=3) AMs measured by flow cytometer. (Student's t-test, p≤0.05).

# Figure S6.

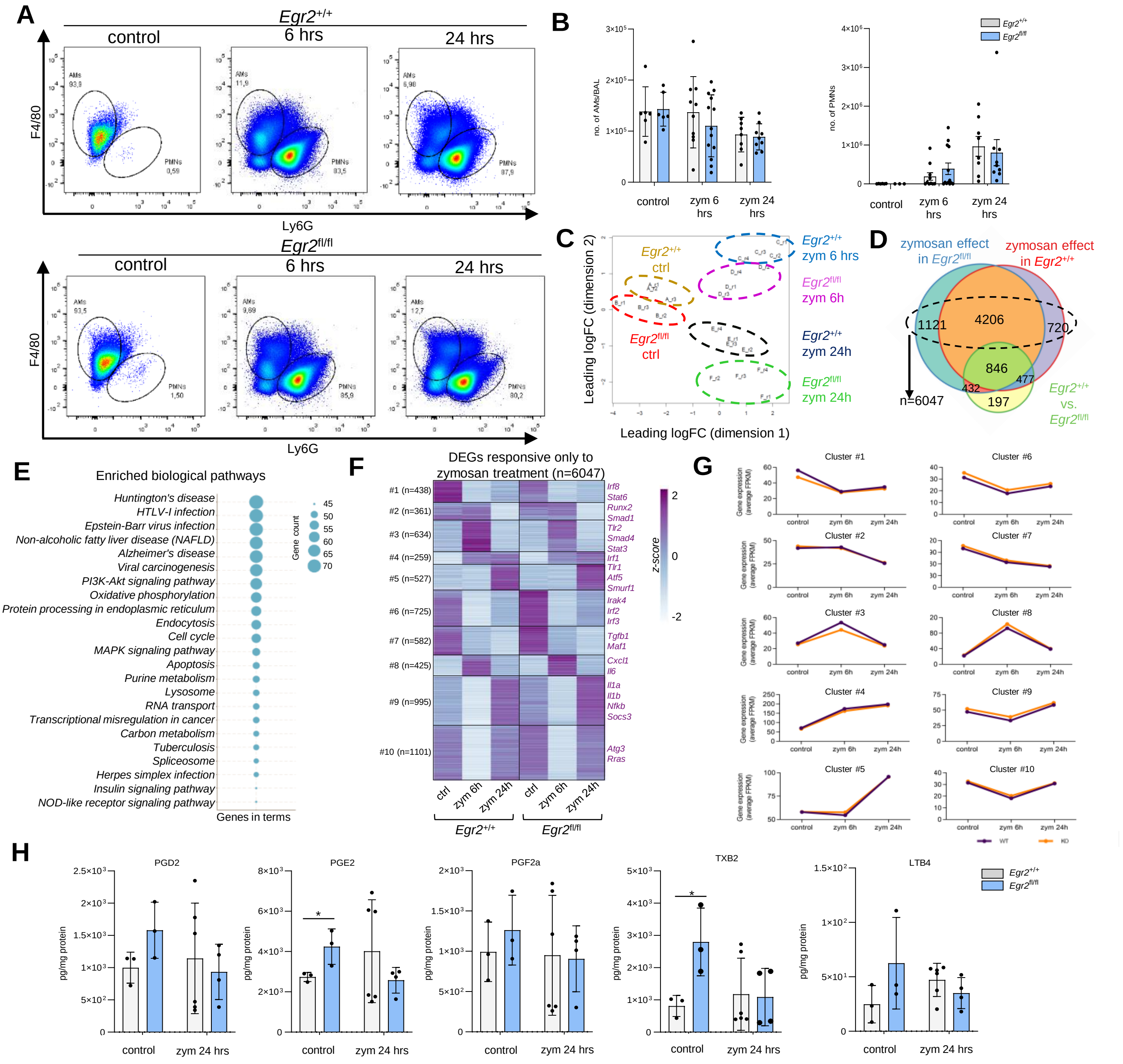

**Figure S6. Characterization of zymosan-induced inflammatory response in alveolar macrophages of *Egr2*$^{fl/fl}$ mice.** **(A)** Representative pseudo color dot plots of CD45$^{+}$ cells from BAL show the ratio of AMs (F4/80$^{+}$ population) and PMNs (Ly6G$^{+}$ population) *in vivo* upon 0, 6 hrs and 24 hrs zymosan treatment. **(B)** The bar graphs show the mean±SEM values of AMs and PMNs in the BAL of *Egr2*$^{+/+}$ ($n_{control}$=6, $n_{6hrs}$=10, $n_{24hrs}$=8) and *Egr2*$^{fl/fl}$ ($n_{control}$=6, $n_{6hrs}$=13, $n_{24hrs}$=9) mice *in vivo* after 0, 6 and 24 hrs zymosan treatment measured by flow cytometry. **(C)** MDS represents the Euclidean distances between the control and zymosan-treated (6 and 24 hours) RNA-seq samples in *Egr2*$^{+/+}$ and *Egr2*$^{fl/fl}$ AMs. **(D)** The Venn diagram represents the overlaps between the changing gene sets defined also in Figure 5 B but highlights only a subset of genes responsive to zymosan treatment (n=6047). **(E)** The table contains the enriched KEGG biological pathways related to the 6047 zymosan responsive genes upon *Egr2*$^{fl/fl}$. The top pathways were selected based on the total number of the target genes and were depicted in that order. **(F)** K-means clustered ($k_n$=10), row-normalized heatmap represents the average gene expression (z-score) of three replicates of control, zymosan 6- and 24-hour treatments for the 6047 genes in both *Egr2*$^{+/+}$ and *Egr2*$^{fl/fl}$ experiments. Representative genes of the clusters were listed on the right. **(G)** Line plots represent the average gene expression values (FPKM) of the clusters' genes (depicted in Figure F) in the control, zymosan 6- and 24-hour treated samples in both *Egr2*$^{+/+}$ (purple) and *Egr2*$^{fl/fl}$ (orange) experiments. **(H)** The amount of different inflammatory lipid mediators in total lung homogenate upon control and zymosan 24 hrs treatment analyzed by LC-MS/MS method (Student's t-test, p≤0.05).

**Figure S7.**

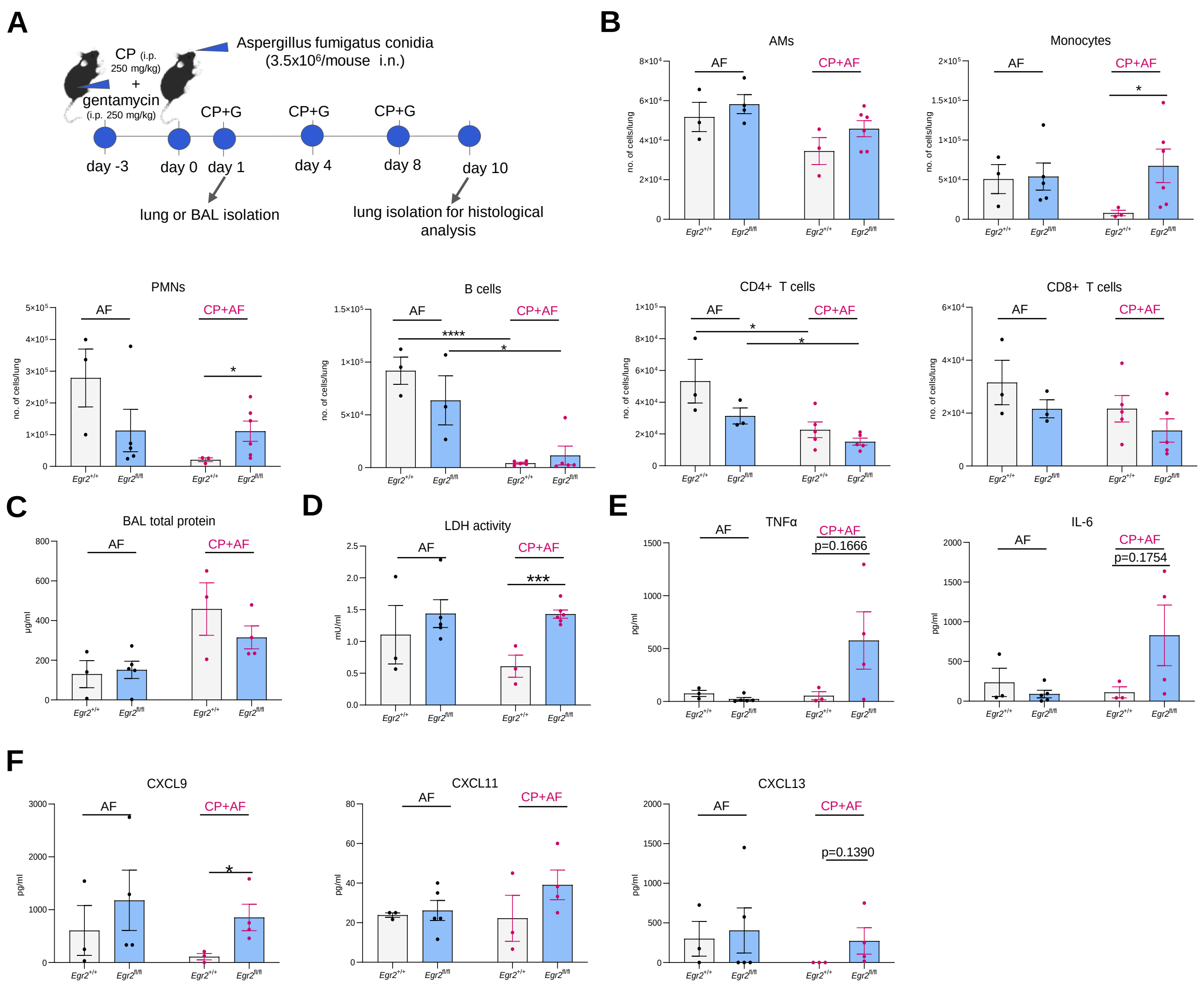


**Figure S7. Comparison of molecular and cellular changes during in vivo Aspergillus fumigatus lung infection with or without cyclophosphamide immunosuppression.**

**(A)** Schematic summary of *in vivo* cyclophospamid (CP) immunosuppression and *Aspergillus fumigatus* (AF) infection based *in vivo* pneumonia model. **(B)** The number of different indicated immune cell types isolated from total lung samples of 24-hour AF-infected and CP pretreated or non-treated *Egr2*$^{+/+}$ (n=3) and *Egr2*$^{fl/fl}$ (n=3 or n=5) mice. **(C)**The bar graphs represent the total protein amounts determined by BCA assay from *Egr2*$^{+/+}$ (n=3) and *Egr2*$^{fl/fl}$ (n=5 or n=4) mice's BALF after 24 hours AF infection with or without CP pretreatment. **(D)** LDH activity in BALF upon 24 hours AF infection with or without CP immunosuppression from *Egr2*$^{+/+}$ (n=3) and *Egr2*$^{fl/fl}$ (n=5 or n=4) mice. **(E-F)** The amount of inflammatory marker proteins (TNFa and IL-6) **(E)** and fibrotic marker cytokines (CXCL9, CXCL11, CXCL13) **(F)** in BALF isolated after 24 hours upon AF infection from only AF-infected or CP pretreated and AF-infected *Egr2*$^{+/+}$ (n=3) and *Egr2*$^{fl/fl}$ (n=5 or n=4) mice measured by ELISA assays. (Student's t-test, p≤0.05).

**Figure S8.**

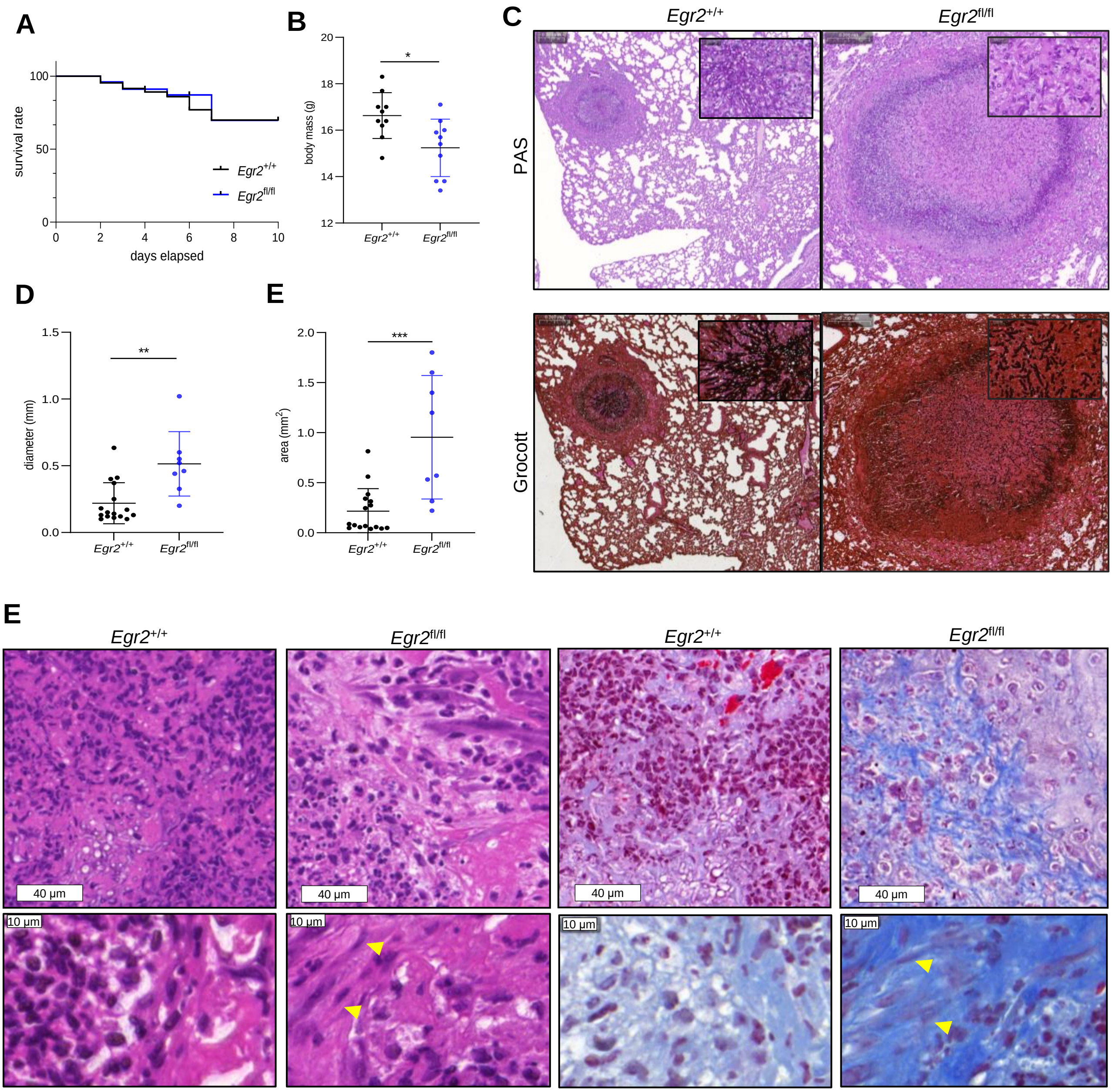


**Figure S8. Patophysiological examination of *Egr2*^fl/fl^ mice during cyclophosphamide immunosuppression and Aspergillus fumigatus infection.**

**(A)** The Kaplan-Meyer survival curve shows the survival rate of *Egr2*$^{+/+}$ (n=15) and *Egr2*$^{fl/fl}$ (n=11) of CP immunosupprimised and AF-infected mice. **(B)** The change in body mass in *Egr2*$^{+/+}$ (n=10) and *Egr2*$^{fl/fl}$ (n=10) mice after *in vivo* intranasal *AF* infection near CP immunosuppression 5-day post infection. **(C)** Representative images of paraffin-embedded and Periodic-Acid-Shiff (PAS) and Grocott (methenamine-silver) stained lung samples of *Egr2*$^{+/+}$ and *Egr2*$^{fl/fl}$ mouse following CP pretreatment and AF exposure via the airway system. The pulmonary aspergillomas manifested and demonstrate the presence of fungal agents (scale bars: 0.2 mm) which are magnified in inserts of each image (scale bar: 0.02 mm). **(D-E)** The mean+/- SEM values of aspegilloma diamteres **(D)** and areas **(E)** 10-day post AF infection and CP immunosuppression measured in representative paraffin-embedded and Grocott stained sections of *Egr2*$^{+/+}$ (n=6) and *Egr2*$^{fl/fl}$ (n=6) lung samples. **(F)** Paraffin-embedded, hematoxylin and eosin (H&E) and Masson's trichrome stained representative images from CP immunosupprimised and AF-infected *Egr2*$^{+/+}$ and *Egr2*$^{fl/fl}$ mouse lungs after 10 days of infection. The images show necrotising inflammation which is composed of predominantly large macrophage-like mononuclear cells with pleomorphic large nuclei or apoptotic cells. In *Egr2*$^{fl/fl}$ samples the persisting inflammatory cells induce large spindle cells, corresponding to activated fibroblasts' morphologies (yellow arrows). Masson staining of such regions shows coarse collagen deposits, reflecting significant fibrosis (blue fibrillar staining). Scale bars: 0.02 (upper) and 0.01 mm (lower).

## Supplemental Methods

### ATAC-seq

*Egr2*$^{+/+}$ and *Egr2*$^{fl/fl}$ murine AMs were isolated by BAL. After the cells were treated with Fc Receptor Blocking Reagent (Miltenyi Biotec, 1:100 dilution) in a total 10 µL volume for 10 minutes, then anti-CD45.2 (BV421 Mouse Anti-Mouse CD45.2 Clone 104, BD Biosciences), anti-F4/80 (APC Rat Anti-Mouse F4/80 Clone T45-2342, BD Biosciences), and anti-CD11c (PE Hamster Anti-Mouse CD11c Clone HL3, BD Biosciences) antibodies were added for 25 minutes at 4 °C in the dark. After centrifugation with the same setting the cell-containing pellet was resuspended in 250 µL staining buffer and 25000 triple-positive cells were sorted. The nuclei were isolated by ATAC-LB (10mM Tris-HCl pH7.4, 10mM NaCl, 3mM MgCl2, 0.1% IGEPAL). Nuclei from cells were applied for tagmentation (Nextera DNA Library Preparation Kit, Illumina) from three biological replicates. After tagmentation, we purified the DNA using the MinElute PCR Purification Kit (Qiagen). The tagmented DNA was amplified by applying Kapa Hifi Hot Start Kit (Kapa Biosystems). The libraries were purified using MinElute PCR Purification Kit (Qiagen). We checked the fragment distribution of libraries with Agilent Bioanalyzer and HiSeq 2500 platform was used for sequencing. We described the analysis of ATAC-seq experiments in the Supplemental Methods.

### ATAC-seq analysis

Raw sequence reads were aligned to the mm10 reference genome assembly with default parameters by using the BWA tool v07.17 and BAM files were generated with SAMtools v1.7 [1, 2]. Peaks were predicted with the callpeak function of MACS2 and their widths were fixed to 200 bp relative to their summits [3]. Artifacts were removed according to the blacklisted genomic regions of ENCODE [4]. Genome coverage (bedgraph) files were generated by HOMER's makeUCSCfile. Differentially opened chromatin regions between the *Egr2*$^{+/+}$ and *Egr2*$^{fl/fl}$ ATAC-seq samples were defined by using DiffBind in R v3.5.1. Peaks that could be

predicted from at least two samples were used for the comparison in which a p≤0.05 cut-off was applied. Read distribution (tag density) heat maps of the different peak sets were carried out by HOMER's annotatePeaks in 10 bp windows within 2 kbp frames relative to the peak summits. The separation of the promoter (promoter-TSS and 5' UTR) and enhancer (introns, intergenic, exons, 3' UTR, and TTS) regions was based on the HOMER's annotatePeaks. Genomic distribution of the peaks was also determined by HOMER's annotatePeaks, where promoter-TSS covers both promoter-TSS (TSS -1000 bp/+100 bp) and 5′ UTRs, and intergenic includes both intergenic and non-coding regions.

**Motif analysis and mapping**

*De novo* motif enrichment analysis was carried out by HOMER's findMotifsGenome and was performed on the summit 200 bp regions of the peaks. The targeted motif lengths were 10, 12, 14, and 16 bp. P-values were calculated by comparing the enrichment within the target regions and that of a random set of regions (background) generated by HOMER. For motif distribution plots, matrices of the enriched motifs were mapped following the order of regions in the tag density heat maps by using the -mbed parameter of HOMER's annotatePeaks in 20 bp windows within 2 kbp frames relative to the peak centers. The top motif score for the different sets of ATAC-seq regions was determined by using the -mscore parameter of HOMER's annotatePeaks.

**RNA-seq**

Total RNA was isolated by TRIZOL (Invitrogen) reagent according to the manufacturer's protocol. The quality of total RNA samples was analyzed using Eukaryotic Total RNA Nano Kit (according to manufacturer's instruction) and Agilent BioAnalyzer. Samples with >7 RNA integrity number [5] were processed for library preparation. Ultra II RNA Sample Preparation Kit (New England BioLabs) was used for library preparation following the manufacturer's protocol. The Poly-A tailed RNA selection was performed by magnetic beads attached with poly-T oligonucleotid. After purification, the mRNA samples were eluted and fragmented at

94°C. Random priming reverse transcription was applied for cDNA generation. After end repair, single adenine base addition and barcode-adaptor ligation the libraries were generated by adapter-specific PCRs. The sequencing run was performed on Illumina NextSeq 500 instrument applying single-end 75-cycle sequencing.

**RNA-seq analysis**

Raw sequence reads were aligned to the mm10 reference genome with HISAT2 v2.1.0 using default parameters and from the BAM files, transcripts were assembled with StringTie v1.3.4d [6, 7]. Bam files were indexed with SAMtools v1.7, then coverage (bedgraph) files were generated by HOMER’s makeUCSCfile program [2, 8]. Expression levels were determined in FPKM values.

The multi-dimensional scaling (MDS) plot was generated by the plotMDS function of the limma package in R [9]. The KEGG biological pathways were predicted with the -go options of HOMER’s annotatePeaks package [10]. K-means clustering of the gene sets (n=1755 and n=6047) was carried out by Cluster 3.0, and the applied similarity metric was the centered correlation [11].

**Integration of RNA-seq and ATAC-seq data**

The assignment of the DARs to the DEGs was based on the linear proximity from the TSSs: gene TSSs were extended to ±100 kbp or to the nearest gene(s) and the changing ATAC-seq peaks overlapped with these extended regions were considered as potential regulatory regions. Overlaps were done with intersectBed commands of BEDtools v2.27.1 [12]. For the distance distribution of the repressed ATAC-seq peaks relative to the repressed genes, the presence of the neighboring genes was not taken into account, only the distance itself (11 bins were defined from <100 to >1000 kbp).

**CUT&RUN**

AMs were isolated from the bronchoalveolar space by lavage in both *Egr2*$^{+/+}$ and *Egr2*$^{fl/fl}$ mice. Cells were then centrifuged with the same settings as before to pellet cells. Cells were

resuspended in 1 mL of freezing media (FBS + 10% dimethyl sulfoxide (DMSO)) and kept at -80 °C until the experiment was performed. On the day of the experiment, cells were recovered from frozen, and 80,000 cells were aliquoted per sample. CUT&RUN was performed with the CUTANA ChIC/CUT&RUN Kit from EpiCypher following the manufacturer's protocol. Briefly cells were bound to concanavalin A beads, the cell membrane was permeabilized and H3K4me3, BRD4, and IgG antibodies were added to their respective samples. Samples were incubated overnight at 4 °C for antibody binding. The following morning the pAG-MNase was added and incubated with the cells for 30 minutes. 100mM $CaCl_2$ is added to each sample to start the pAG-MNase digestion of the chromatin. After the digestion with the pAG-MNase, the reaction is stopped, and DNA is purified using the CUTANA DNA Purification Kit. Sample concentration was checked using the Qubit fluorometer. 1 ng of DNA was used for library preparation. Library preparation was performed using the NEBNext Ultra II DNA Library Prep Kit (New England Biolabs) according to the manufacturer's protocol. Libraries were then assessed using the Agilent Bioanalyzer prior to sequencing. Sequencing was performed using the NextSeq 500 (Illumina) using a Mid Output Kit (150 cycles). 8.5 million reads were obtained per sample. We described the analysis of CUT&RUN experiments in the Supplemental Methods.

**CUT&RUN analysis**

Raw sequence reads were analyzed with default parameters by using the nfcore/cutandrun pipeline v1.1. The reads were aligned to the mm10 reference and spike-in (Escherichia_coli_K_12_MG1655) genome with Bowtie2 v2.4.2. The adapter and quality trimming were performed with Trim Galore! v0.6.6. Bam files were indexed with SAMtools v1.13, then the duplicated reads were marked by using the MarkDuplicates tool of Picard v2.25.7. Peaks were predicted with the callpeak function of MACS2. Genome coverage (bedgraph) files were generated by HOMER's makeUCSCfile. The H3K4me3 signal was measured as Reads Per Kilobase of transcript, per Million mapped reads (RPKM) value on

the downstream 500 bp regions of gene TSSs by using the coverageBed command of BEDtools.

### Visualization

Row-normalized gene expression heatmaps were visualized in R by pheatmap package. Read distribution (tag density) and non-normalized heatmaps were visualized by Java TreeView v1.1.6r4. Histograms, pie- and (stacked) bar charts, box-, violin-, line-, scatter- and volcano plots were plotted by using GraphPad Prism v.9. The area-proportional Venn diagram was generated by Intervene [13]. Genome coverage (bedgraph) files were visualized by IGV v2.4.16 where the replicates that belong together were overlaid on each other (overlay tracks) [14]. Proportional dot plots were visualized by ggplot by using the tidyverse, and ggthemr packages in R.

### Flow cytometry and cell sorting

The cells were incubated with Fc Receptor Blocking Reagent (Miltenyi Biotec) in 1:100 dilution for 10-minute at 4 °C. The samples were stained by specific antibody mix in 0.04 µg/µl dilution for 25-minute at 4 °C in dark. In intracellular staining the cells were fixed and permeabilized using the FoxP3/Transcription Factor Staining Kit's (eBioscienceTM) 1×Fixation/Permeabilization reagent (overnight, 4 °C). Afterwards, cells were washed with 1×Permeabilization/Wash buffer. Intracellular staining was performed in 100 µL 1×Permeabilization/Wash buffer containing the antibody cocktail for 1-hour at 4 °C. Ultimately, cells were washed with Permeabilization buffer and resuspended in MACS buffer. The measurement and sorting were performed with FACS Aria III (BD Biosciences) and Cytek Aurora spectral flow cytometers. We indicated the applied antibodies in Supplemental Methods. FlowJo v.10 software was used for data analysis.

## Applied antibodies for flow cytometry

Anti-CD45.2 (BV421 Mouse Anti-Mouse CD45.2 Clone 104, BD Biosciences and BV750 Clone 30-F11, Biolegend), anti-CD11b (PE-Cy™7 Rat Anti-CD11b Clone M1/70, BD Biosciences and PE-Texas Red Clone M1/70.15, Invitrogen), anti-CD11c (PE Hamster Anti-Mouse CD11c Clone HL3, BD Biosciences and APC-R700 Clone N418, BD Biosciences), anti-F4/80 (APC Rat Anti-Mouse F4/80 Clone T45-2342, BD Biosciences and Pacific Blue Clone BM8, Biolegend), anti-SiglecF (APC-Cy™7 Rat Anti-Mouse Siglec-F Clone E50-2440, BD Biosciences), anti-CLEC7a (BD OptiBuild™ BV711 Rat Anti-Mouse CD369 (Clec7a) Clone 218820, BD Bioscience), anti-Ly6G (PerCP-Cy™5.5 Rat Anti-Mouse Ly-6G Clone 1A8, BD Bioscience and BV510 Clone 1A8, BD Biosciences), anti-CD4 (BUV395 Clone GK1.5, BD Biosciences), anti-CD8 (BUV737, Clone 53-6.7, BD Biosciences), anti-Ly6C (BV605 Clone HK1.4, Biolegend), anti-CD25 (BV650, Clone PC61, BD Biosciences), anti-CD24 (BV711 Clone M1/69, BD Biosciences), anti-CD140a (BV785 Clone APA5, BD Biosciences), anti-CD103 (AF488/FITC Clone 2E7, Invitrogen), anti-CD31 (BB700 Clone MEC 13.3, BD Biosciences), anti-CD64 (PerCP-eFluor 710 Clone X54-5/7.1, Invitrogen), anti-TCR-γδ (APC Clone GL3, Biolegend), anti-MHCII (APC-Cy7 Clone M5/114.15.2, Biolegend), NK1.1 (APC/Fire 810 Clone S17016D, Biolegend), Foxp3 (PE Clone FJK-16s, Invitrogen), anti-CD3 (PE-Cy5 Clone 17A2, Biolegend), anti-CD45R-B220 (PE/Fire 700 Clone RA3-6B2, Biolegend), and anti-CD326 (PE-Cy7 Clone G8.8, Biolegend).

## Measurement of oxygen consumption and extracellular acidification rate

We compared 110,000 AMs isolated by BAL from *Egr2*$^{+/+}$ and *Egr2*$^{fl/fl}$ mice. OCR and changes in ECAR were measured using an XF96 oximeter (Seahorse Biosciences, North Billerica, MA, USA) [15]. Cells were seeded in a 96-well Seahorse assay plate the day prior to the assay. On the day of the assay, the medium was replaced with unbuffered medium 1 hour prior to the assay and then cells were put into a CO2-free incubator to evaporate the remaining CO2. When recording OCR and ECAR, the values were recorded for 5 minutes followed by a 30s

mixing cycle and that cycle was repeated 5 times. Cells received no treatment to record the baseline OCR and ECAR values. Thereafter, cells were treated with inhibitors in the following order: 50 µM Etomoxir, 2 µM Oligomycin and 10 µM Antimycin A. After each inhibitor, a 5-cycle measurement round was performed. At the end of the assay cells were lysed in 1M NaOH, protein content was determined using the BCA Kit (Thermo) and data were normalized to protein content.

The first value after any treatment or the first value of the measurement was omitted. The average of the following 4 reads was used. The OCR and ECAR values after Antimycin A treatment were considered background and were subtracted from all values. Baseline ECAR values were reported after subtracting the background. For the OCR values, Etomoxir sensitive, resistant OCR, oligomycin-sensitive, and resistant OCR were calculated.

**Western Blot**

800,000 *Egr2*$^{+/+}$ and *Egr2*$^{fl/fl}$ AMs were isolated from BAL and whole cell lysates were generated with RIPA buffer. Whole-cell lysates were resolved by electrophoresis in a 4-16% gradient Bis-Tris polyacrylamide gel and transferred to immobilon-P transfer membrane. The membrane was incubated with REVERT Total Protein Stain for 5 minutes before being imaged to get total protein loading. Membranes were then blocked for 1 hr at room temperature before being probed with 1:1000 anti-EGR2 (Abcam, ab108399) antibody overnight in the cold room. The next day membrane was washed with 0.1% TBS-T (5x for 5 minutes) and 1:20000 of anti-Rabbit IgG (R&D Systems, HAF008) secondary antibody was added. Membrane was incubated with the secondary antibody for 1 hr at room temperature then was washed with 0.1% TBS-T (5x for 5 minutes). The membrane was then incubated with SuperSignal West Pico PLUS Chemiluminescent Substrate for 5 minutes before being imaged.

**Generation of EGR2-expressing ESC-derived myeloid progenitor cells**

The EGR2-expressing doxycycline-inducible murine ESCs were cultured in DMEM (Thermo) containing 15% FBS (Thermo), 1000 U/ml LIF (Merck), 50 µg/ml streptomycin, and 100 U/ml

penicillin (Merck) in presence of G418 (200 µg/ml) for chemical reselection of transgenic ESCs [16].

For mesodermal differentiation, embryoid bodies (EBs) were created as described [17] with minor modifications. ESCs were cultured in IMDM (Iscove′s Modified Dulbecco′s Medium; Thermo) with 15% FBS, 200 ng/ml iron-saturated transferrin (Sigma), 4.5 mM monothioglycerol (Sigma) and 50 ng/ml ascorbic acid (Sigma) in slowly rotating Petri dishes for 6 days. Thereafter EBs were harvested with trypsin-EDTA treatment. To obtain myeloid progenitors the dissociated cells were further cultured for 3 days in alpha-MEM medium containing 20% FBS, 50 ng/ml GM-CSF (PeproTech, London, UK), and 50 µM 2-ME (β-mercaptoethanol; Merck) in monolayers. At this stage, 1 µg/ml of doxycycline was added to induce EGR2. Finally, cells were harvested with trypsin/EDTA.

**Real-time quantitative PCR for eRNA and mRNA detection**

Total RNA was isolated by TRIZOL (Invitrogen) reagent according to the manufacturer's protocol. After RNA isolation the High-Capacity cDNA Reverse Transcription Kit (Applied Biosystems) was used for reverse transcription according to the manufacturer's instructions. We applied SYBR Green master mix (BioRad) based RT-qPCR assay for eRNA and mRNA transcript detection. The ΔCt method was used for analysis. We normalized the raw expression values to the *Ppia* expression. mRNA and eRNA primer sequences are provided (Supplemental Table 13).

**Confocal microscopic experiments**

The AMs were isolated by BAL from *Egr2*$^{+/+}$ and *Egr2*$^{fl/fl}$ mice and were purified with CD45 magnetic bead selection (Miltenyi Biotec). For microscopy measurements, 50,000 cells were sub-cultured in 8-well chambered coverslips (Ibidi) and maintained in 300 µl RPMI medium containing 5% penicillin, 5% streptomycin, and 10% FBS until the cells get attached. Next, $2x10^6$ Texas-Red labeled zymosan bioparticles (Invitrogen™, Z2843) were added per one

well. Cells were incubated with the zymosan particles at 37 °C for different time points; 0-, 20-, 60-, and 120-minute. At the specific time point, cells were washed with PBS, and confocal images were recorded by Zeiss LSM 880 microscope (Carl Zeiss, Jena, Germany) using 40×, 1,2 numeric aperture (NA), C-Apochromat water immersion objective. Texas Red fluorescence images and transmitted light images were recorded by using a 543 nm HeNe laser; fluorescence emission was detected between 540-642 nm. The pinhole size was 150 μm resulting in an optical slice thickness of 3.4 μm. The image size was 512×512 pixels (142 μm × 142 μm). The number of the particles in the cells was counted manually and the representative confocal images were processed using the open-source FIJI distribution of ImageJ v2.0.0-rc-69/1.52i.

**Targeted Liquid Chromatography with tandem Mass Spectrometry (LC-MS/MS) analysis of lipid mediators**

The total lungs samples were snap frozen in liquid nitrogen and placed in ice-cold methanol containing deuterated internal standards including $d_4$-$LTB_4$, $d_4$-$PGE_2$, $d_5$-$LXA_4$, $d_5$-MaR1, $d_5$-RvD2, and $d_8$-5-HETE (Cayman Chemical, Ann Arbor, MI). Samples were minced and placed at -80°C for a minimum of 1 hr to allow for protein precipitation. Following centrifugation (13,000 rpm; 10 min; 4°C), supernatants were separated from protein pellets. Pellets were lysed using RIPA buffer (Thermo) and total protein was determined via Pierce BCA assay (Thermo). Supernatants were subjected to solid-phase extraction (SPE) using C18 columns (Biotage) that were preconditioned with successive washes of methanol and water, as described in [18]. Prior to loading onto SPE columns, samples were diluted with water acidified to a pH of approximately 3.5. After washing with hexane, lipid mediators were eluted from the column using methyl formate. The solvent was evaporated under a stream of $N_2$ gas and samples were resuspended in methanol:water (50:50, v/v) prior to analysis by LC-MS/MS.

Samples were analyzed using a high-performance liquid chromatography system (HPLC; Shimadzu, Kyoto, Japan) that was equipped with refrigerated autosampler and a Poroshell reverse-phase C18 column (100 mm x 4.6 mm x 2.7mm; Agilent Technologies) that was held

at 50°C using a column oven. A gradient method was used that started with methanol/water/acetic acid (50:50:0.01 v/v/v) and was ramped to 98:2:0.01 using a constant flow rate of 0.5ml/min. A Qtrap5500 mass spectrometer (AB Sciex, Framingham, MA) was coupled to the HPLC and was operated in negative ionization mode. Data acquisition was accomplished using Analyst software v.1.7. A scheduled multiple reaction monitoring (MRM) method was used to identify lipid mediators based on specific parent ion/daughter ion pairs that were established using synthetic standards for each individual lipid mediator (Cayman Chemical, Ann Arbor, MI). Identification of lipid mediators in the samples was accomplished using Sciex OS-Q v.1.7 by comparing the retention time of mediators in the samples to authentic standards run in parallel, using diagnostic MRM transitions. In selected samples, we also assessed full MS/MS spectra that were acquired utilizing information-dependent enhanced product ion mode scanning. Lipid mediators were quantified by integrating peak areas above the baseline and interpolation based on external standard curves for each individual mediator, followed by accounting for extraction recovery based on internal deuterated standards with similar chromatographic retention times. Levels of lipid mediators were then normalized to the protein content of the tissue taken for extraction. The lower limit of quantification was established for each mediator and was defined as the lowest amount that could be quantified in replicate injections with a coefficient of variation (CV) of less than 20%.

**Mouse Cytokine Array**

We isolated BAL fluid in a total 1 mL volume from *Egr2*$^{+/+}$ and *Egr2*$^{fl/fl}$ mice from the steady-state condition and following 6-hour and 24-hour 300 µg intranasal zymosan treatment. Quantibody® Mouse Cytokine Array 1000 Kit (QAM-CAA-1000) was used for the analysis of cytokine concentrations and the experiment was performed from generated BAL fluid samples by RayBiotech company. Hierarchical clustering of log-scaled protein amount (µg/ml) values was carried out by Cluster 3.0 and the non-scaled heatmap was visualized by JavaTreeView. The applied similarity metric was Euclidean distance, and the clustering method was by single linkage.

**Bronchoalveolar lavage fluid protein analysis**

The total protein level of BALF was determined by BCA assay (Thermo). We measured LDH activity by Lactate Dehydrogenase Activity Assay Kit (Sigma). For the detection of cytokine levels we used duplicate wells and we applied mouse TNFα, IL-6 (Biolegend) and CXCL9, CXCL11, CXCL13 ELISA Kits (Invitrogen). Both were performed following to the manufacturer's instructions.

**Ex vivo Aspergillus fumigatus co-treatment model**

The AMs were isolated by BAL from *Egr2*$^{+/+}$ and *Egr2*$^{fl/fl}$ mice and were purified with CD45 magnetic bead selection (Miltenyi Biotec). We cultured the cells in RPMI medium containing 5% penicillin, 5% streptomycin and 10% FBS. After attachment we added *AF* conidia ($10^6$/well) for $10^5$ AMs. After 1-hour incubation we replaced the medium and we incubated the cells for 6 more hours. After scratching the cells at the first one hour or at the finish of incubation we made decimal dilution series of the lysate. With 1 ml of $10^3$ and $10^4$ dilution members we inoculated nitrate minimal media agar slants and incubated at 37°C for 48 hours. Then we counted the number of CFUs of AF.

**Time-lapse microscopic experiment**

The AMs were isolated by BAL from *Egr2*$^{+/+}$ and *Egr2*$^{fl/fl}$ mice and were purified with CD45 magnetic bead selection (Miltenyi Biotec). For microscopy measurements, 100000 cells were sub-cultured in 6-well plate and maintained 1000 µl RPMI medium containing 5% penicillin, 5% streptomycin, and 10% FBS until the cells get attached. Next, $1x10^6$ *AF* conidia were added per one well for 1 hour, then the media was replaced or just the previously in vivo AF treated cells were plated without any further treatment. Cells were incubated at 37 °C for 10 more hours. Time-lapse images were snaped every 5 second applying Olympus upright microscope (Tokyo, Japan) using 10x, 0.25 NA, plan achromatic objectives (Carl Zeiss, Jena, Germany), and 2-megapixel UVC USB 2.0 camera boards (Asus Computer International,

Fremont, CA, USA). The number of the particles in the cells and the time-point of hypha breakout from cells were counted manually and the representative images were processed using the ImageJ v1.39d software.

### In vivo Aspergillus fumigatus infection model

Mice were housed in plastic cages (425/135/120 mm, 573.75 cm2) in groups of 5-6 with mesh covers according to 2010/63/EU guidelines. Animals were fed with pelleted mouse chow (Purina) and tap water *ad libitum*. Automated room illumination of 12 h light and 12 h dark, and room temperature (22–25 °C) was maintained. Both sexes of *Egr2*$^{+/+}$ and *Egr2*$^{fl/fl}$ mice (3-month-old) were used for all experiments. For the immunosuppression model, we applied intraperitoneal (i.p.) administration of CP (250 mg/kg). The first treatment was performed 3 days before infection and the second one was 1 day after infection. On the same day the animals we administered gentamicin prophylaxis (5-mg/kg body weight) to prevent the bacterial superinfection. The method of inoculation was the instillation of 3.5x10$^{6}$ in 50 μl of a suspension of conidia (7x10$^{7}$/mL) to anesthetized mice [19]. After infection, we waited 11 days to see the symptoms and measure the body weight.

## List of abbreviations

ACK - Ammonium-Chloride-Potassium

AF – Aspergillus fumigatus

AM - alveolar macrophage

AP-1 - Activator Protein 1

ARA - arachidonic acid

ATAC-seq - Assay for Transposase-Accessible Chromatin using sequencing

BAL - bronchoalveolar lavage

BMDM - bone marrow-derived macrophage

BOOP - bronchiolitis obliterans with organizing pneumonia

bp - base pair

BRD4 - Bromodomain Containing 4

BSL2 - biosafety level 2

C/EBP - CCAAT-enhancer-binding Protein

CCL17 - C-C Motif Chemokine Ligand 17

cDNA- complementary DNA

CFU - colony-forming unit

CLR - C-type lectin receptor

CP - cyclophosphamide

CV - coefficient of variation

CXCL11 - C-X-C motif chemokine 11

CSF1 - Colony Stimulating Factor 1

DAR - differentially accessible region

DC – dendritic cell

DEG - differentially expressed gene

DMSO - dimethyl sulfoxide

ECAR - extracellular acidification rate

EDTA - ethylenediaminetetraacetic acid

EGR2 - Early Growth Response 2

eRNA - enhancer RNA

ESC – embryonic stem cell

FBS - Fetal Bovine Serum

FPKM - Fragments Per Kilobase of transcripts per Million mapped reads

GEO - Gene Expression Omnibus

GM-CSF - Granulocyte-Macrophage Colony-Stimulating Factor

H&E - hematoxylin and eosin

HPLC - high-performance liquid chromatography

ID2 - Inhibitor Of DNA Binding 2

IGV - Integrative Genomic Viewer

IL-4- Interleukin 4

IM – interstitial macrophage

kbp - kilobase pair

KEGG - Kyoto Encyclopedia of Genes and Genomes

KO - knock-out

LC-MS/MS - Liquid Chromatography with tandem Mass Spectrometry

LDTF - lineage-determining transcription factor

LTA4H - Leukotriene A4 Hydrolase

LTB4 - leukotriene B4

LysCre - lysozyme-Cre

MDS - multi-dimensional scaling

MFI - median fluorescence intensity

MPO - myeloperoxidase

MRM - multiple reaction monitoring

mRNA - messenger RNA

NA - numeric aperture

NK – natural killer

OCR - oxygen consumption rate

PAMP - pathogen-associated molecular pattern

PAS – periodic acid–Schiff

PBS - Phosphate Buffered Saline

PFA - paraformaldehyde

PGE2 - prostaglandin E2

PI - phosphatidyl-inositol

PMN - polymorphonuclear cell

PPARγ - Peroxisome Proliferator-Activated Receptor Gamma

PRR - pathogen recognition receptor

PU.1 - Purine Rich Box-1

RNA-seq - RNA-sequencing

ROS - reactive oxygen species

RPKM - Reads Per Kilobase of transcript, per Million mapped reads

RT-qPCR - real-time quantitative polymerase chain reaction

RUNX - Runt-Related Transcription Factor

RvE1 - Resolvin E1

SPE - solid-phase extraction

TF – transcription factor

TGF-β - Transforming Growth Factor β

TLR - Toll-like receptor

TSS - transcription start site

TXB2 - thromboxane B2

WT - wild-type

References:


1. Li, H. and R. Durbin, *Fast and accurate short read alignment with Burrows-Wheeler transform.* Bioinformatics, 2009. **25**(14): p. 1754-60.
2. Heinz, S., et al., *Simple combinations of lineage-determining transcription factors prime cis-regulatory elements required for macrophage and B cell identities.* Mol Cell, 2010. **38**(4): p. 576-89.
3. Zhang, Y., et al., *Model-based analysis of ChIP-Seq (MACS).* Genome Biol, 2008. **9**(9): p. R137.
4. Amemiya, H.M., A. Kundaje, and A.P. Boyle, *The ENCODE Blacklist: Identification of Problematic Regions of the Genome.* Sci Rep, 2019. **9**(1): p. 9354.
5. Werner, J.L., et al., *Requisite role for the dectin-1 beta-glucan receptor in pulmonary defense against Aspergillus fumigatus.* J Immunol, 2009. **182**(8): p. 4938-46.
6. Kim, D., et al., *Graph-based genome alignment and genotyping with HISAT2 and HISAT-genotype.* Nat Biotechnol, 2019. **37**(8): p. 907-915.
7. Pertea, M., et al., *StringTie enables improved reconstruction of a transcriptome from RNA-seq reads.* Nat Biotechnol, 2015. **33**(3): p. 290-5.
8. Li, H., et al., *The Sequence Alignment/Map format and SAMtools.* Bioinformatics, 2009. **25**(16): p. 2078-9.
9. Ritchie, M.E., et al., *limma powers differential expression analyses for RNA-sequencing and microarray studies.* Nucleic Acids Res, 2015. **43**(7): p. e47.
10. Kanehisa, M. and S. Goto, *KEGG: kyoto encyclopedia of genes and genomes.* Nucleic Acids Res, 2000. **28**(1): p. 27-30.
11. de Hoon, M.J., et al., *Open source clustering software.* Bioinformatics, 2004. **20**(9): p. 1453-4.
12. Quinlan, A.R. and I.M. Hall, *BEDTools: a flexible suite of utilities for comparing genomic features.* Bioinformatics, 2010. **26**(6): p. 841-2.

13. Khan, A. and A. Mathelier, *Intervene: a tool for intersection and visualization of multiple gene or genomic region sets.* BMC Bioinformatics, 2017. **18**(1): p. 287.
14. Thorvaldsdottir, H., J.T. Robinson, and J.P. Mesirov, *Integrative Genomics Viewer (IGV): high-performance genomics data visualization and exploration.* Brief Bioinform, 2013. **14**(2): p. 178-92.
15. Miko, E., et al., *Methods to Assess the Role of Poly(ADP-Ribose) Polymerases in Regulating Mitochondrial Oxidation.* Methods Mol Biol, 2017. **1608**: p. 185-200.
16. Bencsik, R., et al., *Improved transgene expression in doxycycline-inducible embryonic stem cells by repeated chemical selection or cell sorting.* Stem Cell Res, 2016. **17**(2): p. 228-234.
17. Boto, P., et al., *Zbtb46-dependent altered developmental program in embryonic stem cell-derived blood cell progenitors.* Stem Cells, 2021. **39**(10): p. 1322-1334.
18. Dalli, J., et al., *Lipid Mediator Metabolomics Via LC-MS/MS Profiling and Analysis.* Methods Mol Biol, 2018. **1730**: p. 59-72.
19. Palicz, Z., et al., *In vivo application of a small molecular weight antifungal protein of Penicillium chrysogenum (PAF).* Toxicol Appl Pharmacol, 2013. **269**(1): p. 8-16.

## Supplemental figure legends

### Figure S1. Lack of EGR2 expression in alveolar macrophages of *Egr2*$^{fl/fl}$ mice.

**(A-C)** Scatter plots show the gene expression of *Egr2* in different types of macrophages (source of data: RNA-seq, GSE63340, MiG: microglia, PM: peritoneal macrophage, SM: splenic macrophage, KC: Kuppfer cell, LI M: large intestinal macrophage, SI M: small intestinal macrophage, AM: alveolar macrophage) and monocytes (Mo) (data source: GSE63341) **(B)** the gene expression of the *Egr* gene family members, **(C)** the *Egr2* exons and the *Lyz2* gene in *Egr2*$^{+/+}$ and *Egr2*$^{fl/fl}$ AMs. **(D)** EGR2 Western Blot analysis of *Egr2*$^{+/+}$ and *Egr2*$^{fl/fl}$ AMs. Control and IL-4 treated BMDMs were applied as negative and positive controls.

### Figure S2. Characterization of non-immune and immune cell populations in the lung of *Egr2*$^{fl/fl}$ mice.

**(A)** The applied gating strategy for identify the different cell populations in mouse lung by flow cytometry. **(B-C)** The mean±SEM of number of non-immune **(B)** cells (epithelial cells, endothelial cells, and fibroblasts), and immune **(C)** cells (matured alveolar macrophages (AMs), interstitial macrophages (IMs), Ly6C$^{+}$ and Ly6C$^{-}$ monocytes, neutrophil granulocytes, eosinophil granulocytes, CD103+ dendritic cells (DCs), natural killer (NK) cells, B cells, CD4$^{+}$, CD8$^{+}$, and γδ T cells) of total lung samples from *Egr2*$^{+/+}$ and *Egr2*$^{fl/fl}$ (n=5) mice. (Student's t-test, p≤0.05).

### Figure S3. Characterization of the alveolar macrophages of *Egr2*$^{fl/fl}$ mice.

**(A)** Representative images of paraffin-embedded, H&E- and MPO-stained lung samples of *Egr2*$^{+/+}$ and *Egr2*$^{fl/fl}$ mice (blue arrows: bronchus, red arrows: MPO$^{+}$ cells). **(B)** Representative pseudo color dot plots of CD45$^{+}$ cells of the total lung of *Egr2*$^{+/+}$ and *Egr2*$^{fl/fl}$ mice marked by F4/80 and CD11c antibody. The double-positive population was gated and the expression of SiglecF and CD11b were measured (flow cytometry). **(C)** The mean±SEM percentage of F4/80$^{+}$ and CD11c$^{+}$ cells (AMs) within the CD45$^{+}$ population of total lung samples from *Egr2*$^{+/+}$ (n=15) and *Egr2*$^{fl/fl}$ (n=18) mice. **(D)** The mean±SEM ratio of different subpopulations of AMs based on SigelcF and CD11b expression in the lung of *Egr2*$^{+/+}$ (n=5) and *Egr2*$^{fl/fl}$ (n=7) mice. **(E)** The box plots show the MFI of AM markers in CD45$^{+}$ cells isolated from BAL and measured by flow cytometric method in *Egr2*$^{+/+}$ (n=5) and *Egr2*$^{fl/fl}$ (n=10) samples. **(F)** The mean±SEM

OCR of *Egr2*$^{+/+}$ (n=7) and *Egr2*$^{fl/fl}$ (n=5) AMs isolated from BAL and measured by Agilent Seahorse Analyzer. **(G)** The bar graphs represent the mean±SEM OCR values after etomoxir, oligomycin, and antimycin A treatment in *Egr2*$^{+/+}$ (n=7) and *Egr2*$^{fl/fl}$ (n=5) AMs isolated from BAL and measured by Agilent Seahorse Analyzer. **(H)** The mean±SEM ECAR values of *Egr2*$^{+/+}$ (n=4) and *Egr2*$^{fl/fl}$ (n=4) AMs (Agilent Seahorse Analyzer measurement). **(I)** Mean OCR and ECAR values were plotted against each other. (Student's t-test, p≤0.05).

**Figure S4. The possibility of EGR2 autoregulation in alveolar macrophages.**

**(A)** The correlation heatmap represents the similarity among ATAC-seq replicates. **(B)** IGV snapshot of *Egr2* locus, the common enhancers (yellow) in AMs and BMDMs or AM-specific (blue) enhancers based on ATAC-seq and annotated EGR binding motifs (red). **(C)** Positions of enhancers in *Egr2* locus and their presence in BMDMs or AMs. **(D)** The mean±SEM values of normalized eRNA levels of EGR motif-containing common (E12, E13, and E20; yellow) and AM-specific (E14.2, E24.1, and E24.2; blue) enhancers in *Egr2*$^{+/+}$ (n=3) and *Egr2*$^{fl/fl}$ (n=5) AMs measured by RT-qPCR (Student's t-test, p≤0.05).

**Figure S5. Characterization of phagocytosis of alveolar macrophages of *Egr2*$^{fl/fl}$ mice.**

**(A, B, C)** Representative pseudo color dot plots of CD45$^{+}$ and F4/80$^{+}$ *Egr2*$^{+/+}$ and *Egr2*$^{fl/fl}$ AMs after 1 hr or 3 hrs pHrodo-Red conjugated E.coli **(A)**, S.aureus **(B)** or dextran **(C)** treatment (flow cytometry). **(D, E, F)** Bar graphs represent the mean±SEM percentage of non-phagocytotic (pHrodo$^{-}$) and phagocytotic (pHrodo$^{+}$) AMs after 1 hr and 3 hrs pHrodo-Red conjugated E.coli **(D)**, S.aureus **(E)** or dextran **(F)** treatment in *Egr2*$^{+/+}$ (n=4) and *Egr2*$^{fl/fl}$ (n=3) AMs measured by flow cytometer. (Student's t-test, p≤0.05).

**Figure S6. Characterization of zymosan-induced inflammatory response in alveolar macrophages of *Egr2*$^{fl/fl}$ mice.**

**(A)** Representative pseudo color dot plots of CD45$^{+}$ cells from BAL show the ratio of AMs (F4/80$^{+}$ population) and PMNs (Ly6G$^{+}$ population) *in vivo* upon 0, 6 hrs and 24 hrs zymosan treatment. **(B)** The bar graphs show the mean±SEM values of AMs and PMNs in the BAL of *Egr2*$^{+/+}$ ($n_{control}$=6, $n_{6hrs}$=10, $n_{24hrs}$=8) and *Egr2*$^{fl/fl}$ ($n_{control}$=6, $n_{6hrs}$=13, $n_{24hrs}$=9) mice *in vivo* after 0, 6 and 24 hrs zymosan treatment measured by flow cytometry. **(C)** MDS represents the Euclidean distances between the control and zymosan-treated (6 and 24 hours) RNA-seq samples in *Egr2*$^{+/+}$ and *Egr2*$^{fl/fl}$ AMs. **(D)** The Venn diagram represents the overlaps between the changing gene sets defined also in Figure 5 B but highlights only a subset of genes responsive to zymosan treatment (n=6047). **(E)** The table contains the enriched KEGG biological pathways related to the 6047 zymosan responsive genes upon *Egr2*$^{fl/fl}$. The top pathways were selected based on the total number of the target genes and were depicted in that order. **(F)** K-means clustered ($k_n$=10), row-normalized heatmap represents the average gene expression (z-score) of three replicates of control, zymosan 6- and 24-hour treatments for the 6047 genes in both *Egr2*$^{+/+}$ and *Egr2*$^{fl/fl}$ experiments. Representative genes of the clusters were listed on the right. **(G)** Line plots represent the average gene expression values (FPKM) of the clusters' genes (depicted in Figure F) in the control, zymosan 6- and 24-hour treated samples in both *Egr2*$^{+/+}$ (purple) and *Egr2*$^{fl/fl}$ (orange) experiments. **(H)** The amount of different inflammatory lipid mediators in total lung homogenate upon control and zymosan 24 hrs treatment analyzed by LC-MS/MS method (Student's t-test, p≤0.05).

**Figure S7. Comparison of molecular and cellular changes during in vivo Aspergillus fumigatus lung infection with or without cyclophosphamide immunosuppression.**

**(A)** Schematic summary of *in vivo* cyclophospamid (CP) immunosuppression and *Aspergillus fumigatus* (AF) infection based *in vivo* pneumonia model. **(B)** The number of different indicated

immune cell types isolated from total lung samples of 24-hour AF-infected and CP pretreated or non-treated *Egr2*$^{+/+}$ (n=3) and *Egr2*$^{fl/fl}$ (n=3 or n=5) mice. **(C)**The bar graphs represent the total protein amounts determined by BCA assay from *Egr2*$^{+/+}$ (n=3) and *Egr2*$^{fl/fl}$ (n=5 or n=4) mice's BALF after 24 hours AF infection with or without CP pretreatment. **(D)** LDH activity in BALF upon 24 hours AF infection with or without CP immunosuppression from *Egr2*$^{+/+}$ (n=3) and *Egr2*$^{fl/fl}$ (n=5 or n=4) mice. **(E-F)** The amount of inflammatory marker proteins (TNFa and IL-6) **(E)** and fibrotic marker cytokines (CXCL9, CXCL11, CXCL13) **(F)** in BALF isolated after 24 hours upon AF infection from only AF-infected or CP pretreated and AF-infected *Egr2*$^{+/+}$ (n=3) and *Egr2*$^{fl/fl}$ (n=5 or n=4) mice measured by ELISA assays. (Student's t-test, $p \leq 0.05$).

**Figure S8. Patophysiological examination of *Egr2*$^{fl/fl}$ mice during cyclophosphamide immunosuppression and Aspergillus fumigatus infection.**

**(A)** The Kaplan-Meyer survival curve shows the survival rate of *Egr2*$^{+/+}$ (n=15) and *Egr2*$^{fl/fl}$ (n=11) of CP immunosupprimised and AF-infected mice. **(B)** The change in body mass in *Egr2*$^{+/+}$ (n=10) and *Egr2*$^{fl/fl}$ (n=10) mice after *in vivo* intranasal *AF* infection near CP immunosuppression 5-day post infection. **(C)** Representative images of paraffin-embedded and Periodic-Acid-Shiff (PAS) and Grocott (methenamine-silver) stained lung samples of *Egr2*$^{+/+}$ and *Egr2*$^{fl/fl}$ mouse following CP pretreatment and AF exposure via the airway system. The pulmonary aspergillomas manifested and demonstrate the presence of fungal agents (scale bars: 0.2 mm) which are magnified in inserts of each image (scale bar: 0.02 mm). **(D-E)** The mean+/- SEM values of aspegilloma diamteres **(D)** and areas **(E)** 10-day post AF infection and CP immunosuppression measured in representative paraffin-embedded and Grocott stained sections of *Egr2*$^{+/+}$ (n=6) and *Egr2*$^{fl/fl}$ (n=6) lung samples. **(F)** Paraffin-embedded, hematoxylin and eosin (H&E) and Masson's trichrome stained representative images from CP immunosupprimised and AF-infected *Egr2*$^{+/+}$ and *Egr2*$^{fl/fl}$ mouse lungs after 10 days of infection. The images show necrotising inflammation which is composed of predominantly large macrophage-like mononuclear cells with pleomorphic large nuclei or apoptotic cells. In *Egr2*$^{fl/fl}$ samples the persisting inflammatory cells induce large spindle cells, corresponding to activated fibroblasts' morphologies (yellow arrows). Masson staining of such regions shows coarse collagen deposits, reflecting significant fibrosis (blue fibrillar staining). Scale bars: 0.02 (upper) and 0.01 mm (lower).
