# Supplementary material for: EGR2 is an epigenomic regulator of phagocytosis and antifungal immunity in alveolar macrophages
Source: JCI Insight. 2024 Sep 10;9(17):e164009. doi: 10.1172/jci.insight.164009 (PMC11385099; doi:10.1172/jci.insight.164009)

Signal

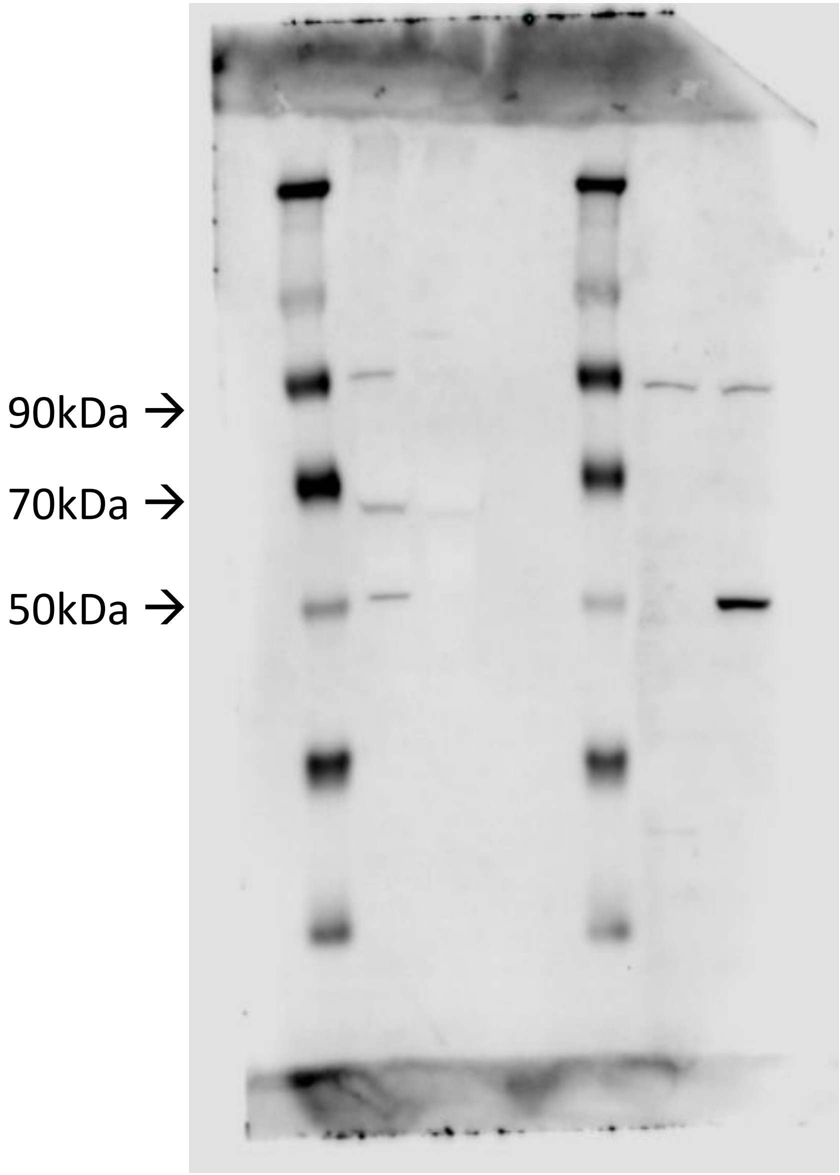

Lane 1: Ladder
Lane 2: WT Alveolar Macrophage
Lane 3: EGR2 KO Alveolar Macrophage
Lane 4: empty
Lane 5: Ladder
Lane 6: BMDM CTR
Lane 7: BMDM 24h IL-4

Total Protein

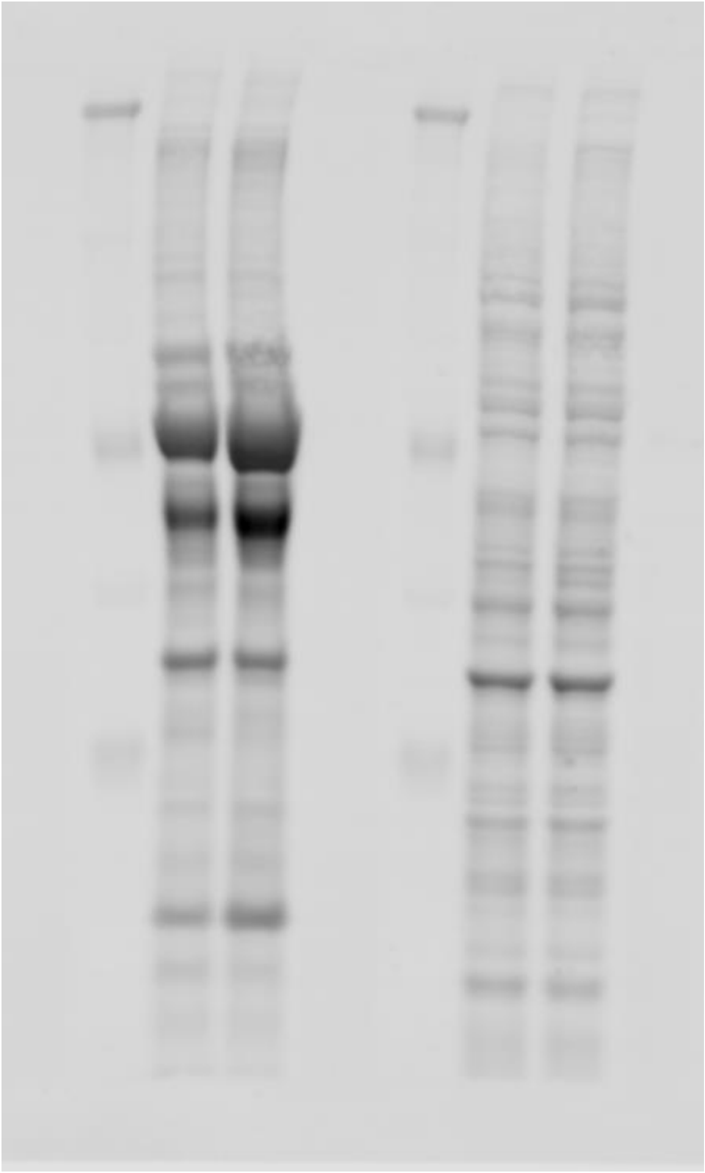

Supplement: Unedited blot and gel images [file jciinsight-9-164009-s049.pdf]
